# A Xenobiotic Detoxification Pathway through Transcriptional Regulation in Filamentous Fungi

**DOI:** 10.1128/mBio.00457-18

**Published:** 2018-07-17

**Authors:** Hyunkyu Sang, Jonathan P. Hulvey, Robert Green, Hao Xu, Jeongdae Im, Taehyun Chang, Geunhwa Jung

**Affiliations:** a Stockbridge School of Agriculture, University of Massachusetts, Amherst, Massachusetts, USA; b Department of Biology, Eastern Connecticut State University, Willimantic, Connecticut, USA; c School of Biosciences, University of Birmingham, Birmingham, United Kingdom; d Department of Civil Engineering, Kansas State University, Manhattan, Kansas, USA; e School of Ecology and Environmental System, Kyungpook National University, Sangju, South Korea; Cornell University

**Keywords:** ATP-binding cassette transporter, fungus-specific transcription factor, multidrug resistance, xenobiotic detoxification, cytochrome P450, *Sclerotinia homoeocarpa*

## Abstract

Fungi are known to utilize transcriptional regulation of genes that encode efflux transporters to detoxify xenobiotics; however, to date it is unknown how fungi transcriptionally regulate and coordinate different phases of detoxification system (phase I, modification; phase II, conjugation; and phase III, secretion). Here we present evidence of an evolutionary convergence between the fungal and mammalian lineages, whereby xenobiotic detoxification genes (phase I coding for cytochrome P450 monooxygenases [CYP450s] and phase III coding for ATP-binding cassette [ABC] efflux transporters) are transcriptionally regulated by structurally unrelated proteins. Following next-generation RNA sequencing (RNA-seq) analyses of a filamentous fungus, Sclerotinia homoeocarpa, the causal agent of dollar spot on turfgrasses, a multidrug resistant (MDR) field strain was found to overexpress phase I and III genes, coding for CYP450s and ABC transporters for xenobiotic detoxification. Furthermore, there was confirmation of a gain-of-function mutation of the fungus-specific transcription factor S. homoeocarpa XDR1 (ShXDR1), which is responsible for constitutive and induced overexpression of the phase I and III genes, resulting in resistance to multiple classes of fungicidal chemicals. This fungal pathogen detoxifies xenobiotics through coordinated transcriptional control of CYP450s, biotransforming xenobiotics with different substrate specificities and ABC transporters, excreting a broad spectrum of xenobiotics or biotransformed metabolites. A Botrytis cinerea strain harboring the mutated ShXDR1 showed increased expression of phase I (*BcCYP65*) and III (*BcatrD*) genes, resulting in resistance to fungicides. This indicates the regulatory system is conserved in filamentous fungi. This molecular genetic mechanism for xenobiotic detoxification in fungi holds potential for facilitating discovery of new antifungal drugs and further studies of convergent and divergent evolution of xenobiotic detoxification in eukaryote lineages.

## IMPORTANCE

Emerging multidrug resistance (MDR) in pathogenic filamentous fungi is a significant threat to human health and agricultural production. Understanding mechanisms of MDR is essential to combating fungal pathogens; however, there is still limited information on MDR mechanisms conferred by xenobiotic detoxification. Here, we report for the first time that overexpression of phase I drug-metabolizing monooxygenases (cytochrome P450s) and phase III ATP-binding cassette efflux transporters is regulated by a gain-of-function mutation in the fungus-specific transcription factor in the MDR strains of the filamentous plant-pathogenic fungus Sclerotinia homoeocarpa. This study establishes a novel molecular mechanism of MDR through the xenobiotic detoxification pathway in filamentous fungi, which may facilitate the discovery of new antifungal drugs to control pathogenic fungi.

## INTRODUCTION

All organisms are constantly exposed to xenobiotics, such as toxic compounds, and defend against these compounds through the coordinated action of metabolizing enzymes and efflux transporter proteins. In mammals and insects, xenobiotic-induced transcriptional regulation of the enzymes and transporters has been established (1, 2). Multiple nuclear receptors have been identified in mammalian systems that contribute to this regulatory response. For example, the nuclear receptor pregnane X receptor (PXR), together with retinoid X receptor (RXR), directly binds structurally unrelated xenobiotics and upregulates transcription of metabolizing enzymes and efflux transporters (3). These include phase I metabolizing enzymes (cytochrome P450s, cytochrome P proteins [CYPs], and monooxygenases) that catalyze xenobiotics and endobiotics mainly through hydroxylation/oxidation reactions (4, 5), phase II conjugating enzymes (UDP-glucuronosyltransferase, sulfotransferase, and glutathione *S*-transferase) that add polar molecules onto compounds, producing water-soluble, nontoxic metabolites (2, 6), and the phase III secretion system, consisting of ATP-binding cassette (ABC) and other transmembrane transporters that actively export parent and/or metabolized compounds across the cytoplasmic membrane (7). Unlike the detailed studies in mammalian systems, the coordinated transcriptional regulation of the three-phase system of these xenobiotic detoxification genes in fungi has not been characterized.

Most studies of xenobiotic detoxification in fungi have focused on antifungal drugs (fungicides) and efflux activities by ABC or major facilitator superfamily (MFS) transporters. The expression of efflux transporters is mainly regulated by fungal zinc-cluster transcription factors (TFs [Zn_2_Cys_6_]) (3). Interestingly, Zn_2_Cys_6_ TF Pdr1 orthologs from the model fungus Saccharomyces cerevisiae and human opportunistic pathogenic fungus *Candida glabrata* were suggested to represent functional analogues of PXR because of the similarities of the xenobiotic signaling by binding chemically diverse xenobiotics and activating the expression of efflux transporters. Although protein sequences and structural folds of PDR1 orthologs and PXR are clearly distinct, they share general architectural similarities, such as DNA binding domains (DBDs) containing cysteines, large ligand binding domains, and short activation domains. Therefore, it is reasonable to propose that emergence of nuclear receptor-like ligand-dependent gene regulatory mechanisms occurred early during eukaryotic evolution, but it has not been determined whether PDR1 orthologs and PXR evolved convergently or divergently (8). Furthermore, in contrast to PXR regulatory mechanisms, Pdr1 orthologs activate expression of phase III efflux transporters only, but not phase I and II metabolizing enzymes during metabolism of xenobiotics.

The emergence of multidrug resistance (MDR) in both pathogenic fungi and human cancers has had a profound impact on human health. The human PXR has been suggested to play an important role in MDR in cancers because of the upregulation of its expression in different cancer cells, its great flexibility in recognizing structurally diverse compounds, and its role as a master regulator of a three-phase detoxification system (9). However, the direct function of upregulation or mutation(s) in PXR to MDR in human cancers *in vivo* has not been understood (8). In human-pathogenic yeasts, molecular mechanisms of MDR have been characterized through functional studies of activating mutation(s) in Zn_2_Cys_6_ transcription factors (PDR1 in C. glabrata or Tac1 in C. albicans), which confer upregulation of efflux transporters resulting in MDR (10–12). The recent studies of gene activation by PDR1 and the Gal11p/MED15 mediator led to the finding of a compound (iKIX1) that disrupts the interaction between PDR1 and mediator and inhibits MDR in C. glabrata strains (13). MDR in filamentous human- and plant-pathogenic fungi is a growing issue to threaten human health and agricultural production (14), but there is a lack of information on MDR mechanisms conferred by the xenobiotic detoxification system for filamentous pathogenic fungi due to limited evolutionary conservation of the subfamily of transcription factors involved in MDR regulation between Saccharomycotina (ascomycete yeasts) and Pezizomycotina (filamentous ascomycete fungi).

Sclerotinia homoeocarpa is a filamentous ascomycete fungus and the causal agent of dollar spot on turfgrasses. Due to repeated application of multiple fungicides, S. homoeocarpa populations have developed resistance to benzimidazole, dicarboximide, demethylation inhibitor (DMI), and succinate dehydrogenase inhibitor (SDHI) fungicides (15–18). Recently, the emergence of MDR populations has been reported in S. homoeocarpa (19). In this study, next-generation RNA sequencing (RNA-seq) analyses of an MDR field strain of S. homoeocarpa revealed enrichment of CYPs and ABC efflux transporters associated with phases I and III of a xenobiotic detoxification system, respectively. We show here that detoxification of different classes of chemicals in S. homoeocarpa occurs through multiple CYPs and ABC transporters, which are coordinately regulated by a putative xenobiotic detoxification regulator (ShXDR1). Furthermore, our results demonstrate a novel gain-of-function dominant mutation (M853T) in S. homoeocarpa XDR1 (ShXDR1^M853T^), identified from the MDR field strain, is responsible for overexpression of both phase I and III genes, leading to MDR. The mechanistic similarity in ways of transcriptionally regulating metabolizing enzymes and efflux transporters for xenobiotic detoxification in fungi and animals suggests new evolutionary insight into xenobiotic detoxification in eukaryotes.

## RESULTS

### Differential gene expression between S. homoeocarpa drug-sensitive and MDR field strains.

**FIG 1:**
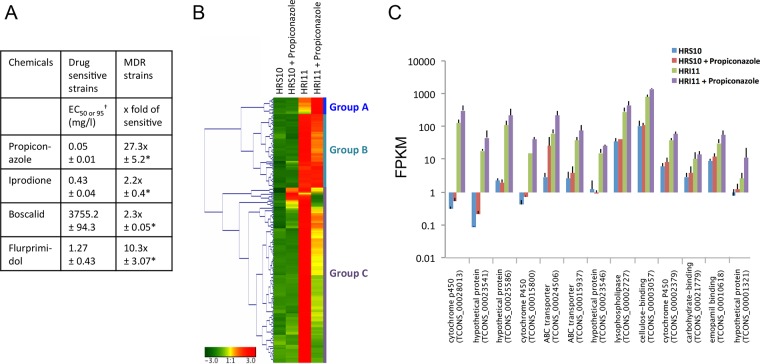
RNA-seq of the drug-sensitive strain HRS10 and multidrug-resistant (MDR) strain HRI11 in the absence and presence of the DMI fungicide propiconazole. (A) *In vitro* sensitivity of drug-sensitive and MDR strains to fungicides and plant growth regulator. Mean values from five MDR strains and five sensitive strains are shown. EC_50_ values for propiconazole, iprodione, and flurprimidol and EC_95_ (†) values for boscalid are shown for sensitive strains. The fold values of EC_50_ and EC_95_ values for sensitive strains are shown for MDR strains. Significant differences from mean values of sensitive strains are indicated by asterisks: *, *P* < 0.001. (B) Expression patterns for 208 genes constitutively overexpressed (log_2_ fold change of ≥1.5 and *P* < 0.05) in strain HRI11, compared to the genes in strain HRS10, before and after treatment with propiconazole. Red indicates higher relative expression, and green indicates lower relative expression. Three major groups of genes, A (*n =* 13), B (*n =* 57), and C (*n =* 138), were clustered based on the analysis of hierarchical clustering of gene expression patterns. (C) FPKM of 13 genes from group A in strains HRS10 and HRI11 before and after exposure to propiconazole.

To determine the MDR mechanism in S. homoeocarpa, *in vitro* sensitivity tests of five MDR field strains and five drug-sensitive field strains to different classes of fungicides (19) and the plant growth regulator (gibberellin biosynthesis inhibitor) were conducted in this study. The group of MDR strains exhibited significant resistance to propiconazole, iprodione, boscalid, and flurprimidol (*P* < 0.001) compared to the group of drug-sensitive strains (Fig. 1A). Samples from one of the MDR strains, HRI11, and one of the drug-sensitive strains, HRS10, before and after exposure to propiconazole (0.1 µg ml^−1^) for 1 h were used for next-generation RNA sequencing (RNA-seq) to profile genome-wide patterns of differential gene expression between these two strains.

RNA-seq analysis identified a total of 28,914 transcripts, corresponding to 12,008 total genes, under the four conditions for both strains. As described previously by Hulvey et al. (20), the strains HRS10 and HRI11 are putative clones. HRI11’s genome was chosen to be the reference because its draft genome is more fully complete. To verify the similarity between the genomes of the two strains, the transcripts were aligned to the whole genomes by BLASTN using an E value set to 0.001. Among the 28,914 transcripts, 28,901 transcripts mapped to HRI11’s genome, and 28,640 mapped to HRS10’s genome. Volcano plots of RNA-seq data indicated that among the 9,499 genes used in the analysis, 55, 7, 94, and 116 genes were significantly downregulated and 59, 11, 208, and 152 genes were significantly upregulated in HRS10 versus HRS10 plus propiconazole, HRI11 versus HRI11 plus propiconazole, HRS10 versus HRI11, and HRS10 plus propiconazole versus HRI11 plus propiconazole, respectively (see Fig. S1 in the supplemental material).

10.1128/mBio.00457-18.2 FIG S1  Volcano plots showing FPKM log_2_ fold change versus log_10_ (*P* value) in RNA-seq data from Sclerotinia homoeocarpa drug-sensitive strain HRS10 and multidrug-resistant strain HRI11 under the conditions of (A) HRS10 versus HRS10 after treatment (+) with propiconazole, (B) HRI11 versus HRI11 plus propiconazole, (C) HRS10 and HRI11, and (D) HRS10 plus propiconazole versus HRI11 plus propiconazole. Using a *P* value of 0.05 as the cutoff threshold, genes not significantly changed are depicted as black circles, and genes significantly changed are indicated as red circles. Download FIG S1, TIF file, 1.9 MB. Copyright © 2018 Sang et al. 2018 Sang et al. This content is distributed under the terms of the Creative Commons Attribution 4.0 International license.

Two hundred eight genes in the MDR strain showed significantly higher expression (log_2_ fold change of ≥1.5 at *P* < 0.05) than the genes in the sensitive strain. The 208 genes were clustered into three major groups by the analysis of hierarchical clustering of gene expression patterns (Fig. 1B). Group A consisted of 13 genes that showed constitutive overexpression and propiconazole-induced expression in the MDR strain. This group includes three genes coding for cytochrome P450s (CYP450s) and two genes coding for ABC transporters (*ShPDR1* and *ShatrD*) previously confirmed for indirect or direct involvement in fungicide resistance (19, 20). These CYP450s and ABC transporters belong to phases I and III in the xenobiotic detoxification pathway, respectively. The group also includes one lysophospholipase (phospholipase B1) gene, one cellulose-binding protein gene, one carbohydrate-binding protein gene, one emopamil-binding protein (EBP) gene, and four hypothetical protein genes (Fig. 1C). We found that the majority of group A genes encode proteins involved in oxidation reduction process (GO:0055114) and transmembrane transport (GO:0055085). Group B consisted of 57 genes that were constitutively overexpressed in the MDR strain but showed no difference or less reduction of expression in response to propiconazole compared to the genes in the sensitive strain. This group includes one dioxygenase gene and one MFS transporter gene that are related to xenobiotic detoxification. The majority of group B genes are related to oxidation reduction process and proteolysis (GO:0006508). Group C consisted of 138 genes that displayed constitutive overexpression and downregulated expression after treatment of propiconazole in the MDR strain. This group includes xenobiotic detoxification-related genes that include one cytochrome 450 gene, one flavin-binding monooxygenase gene, one glutathione *S*-transferase gene, and three MFS transporter genes. Many genes in this group were found to be associated with single organism cellular process (GO:0044763), oxidation reduction process, and cellular nitrogen compound biosynthetic process (GO:0044271).

### Xenobiotic detoxification through phase I cytochrome P450s and phase III ABC transporters.

**FIG 2:**
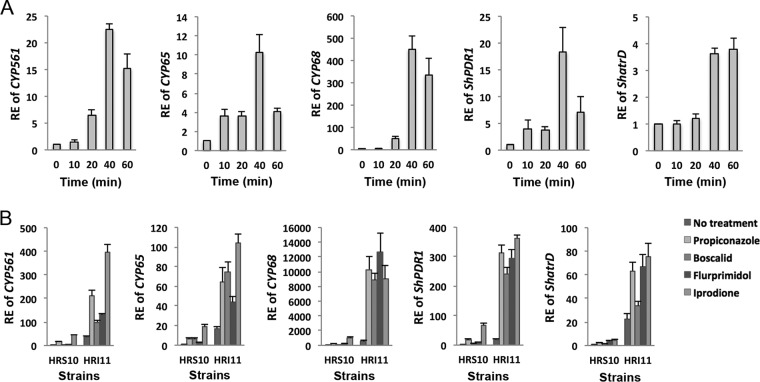
Expression patterns of phase I CYP450s and phase III transporters in response to fungicides and plant growth regulator. (A) Relative expression (RE) of phase I genes (*CYP561*, *CYP65*, and *CYP68*) and phase III genes (*ShPDR1* and *ShatrD*) before and after exposure to propiconazole (1 µg ml^−1^) for 10, 20, 40, and 60 min in strain HRS10. (B) RE of phase I and III genes before and after exposure to propiconazole (1 µg ml^−1^), boscalid (100 µg ml^−1^), flurprimidol (10 µg ml^−1^), and iprodione (10 µg ml^−1^) for 40 min in strains HRS10 and HRI11.

To validate whether overexpression of genes coding for three phase I enzymes (*CYP561*, *CYP65*, and *CYP68*) and two phase III transporters (*ShPDR1* and *ShatrD*) selected from group A is responsible for xenobiotic detoxification and MDR phenotypes, expression of these phase I and III genes was quantified at different propiconazole exposure times and in response to different classes of fungicides and one plant growth regulator. In addition, the overexpression mutants of phase I and III genes from a drug-sensitive strain HRS10 were generated. The expression of *CYP561*, *CYP65*, *CYP68*, *ShPDR1*, and *ShatrD* was induced after treatment of propiconazole between 20 and 40 min in the sensitive strain, HRS10 (Fig. 2A), whose transcriptional response was rapid and transient. These five genes were also constitutively overexpressed in the MDR strain and induced by propiconazole, boscalid, flurprimidol, and iprodione in both sensitive and MDR strains (Fig. 2B).

**FIG 3:**
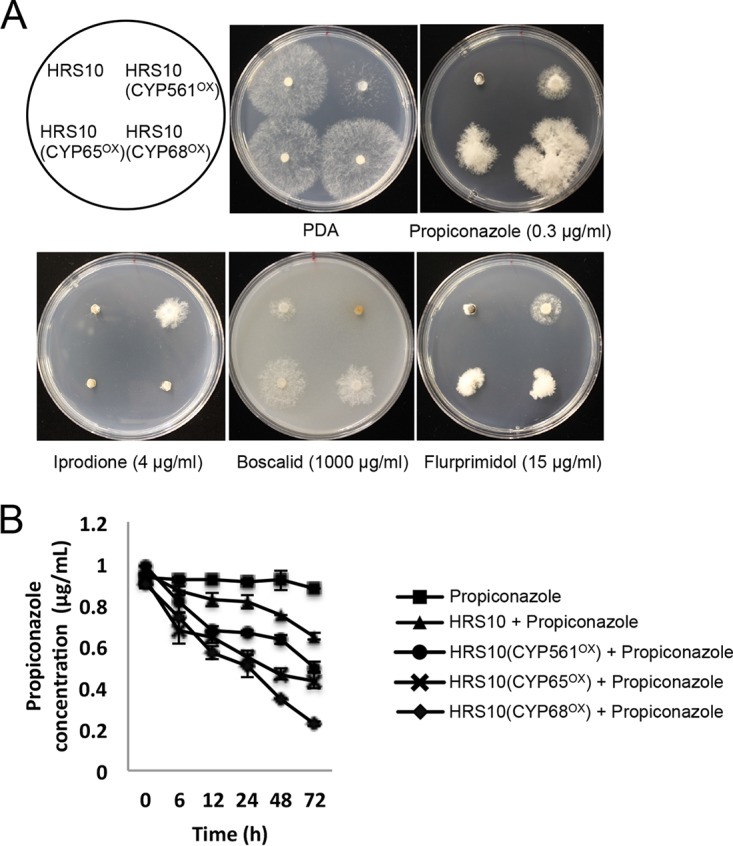
Phase I CYP450s are involved in xenobiotic detoxification and multidrug resistance. (A) Sensitivity of strain HRS10 and mutants overexpressing CYP561, CYP65, and CYP68 to fungicides and plant growth regulator. (B) Propiconazole biotransformation rate of strain HRS10 and mutants overexpressing CYP561, CYP65, and CYP68 by HPLC analysis.

**FIG 4:**
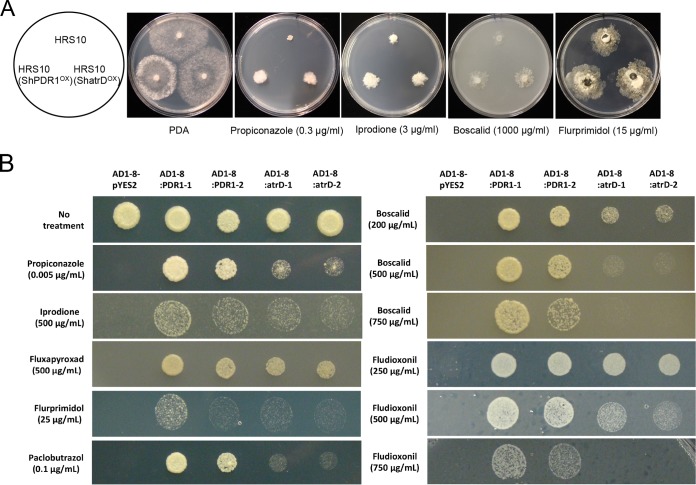
Phase III transporters are involved in xenobiotic detoxification and multidrug resistance. (A) Sensitivity of strain HRS10 and mutants overexpressing ShPDR1 and ShatrD to fungicides and plant growth regulator. (B) Heterologous expression of ABC transporter *ShPDR1* or *ShatrD* in a drug-hypersensitive yeast mutant (AD1–8). Sensitivity tests of the yeast strains were performed on Bacto yeast nitrogen base (YNB) agar medium lacking uracil, containing 2% galactose and amended with different classes of fungicides and plant growth regulator. The sensitivity assays of strains AD1–8-pYES2 and AD1–8:PDR1 with propiconazole, iprodione, boscalid, flurprimidol, and paclobutrazol were reported in previous studies (19, 49).

Mutants with CYP561, CYP65, and CYP68 overexpressed exhibited significantly increased levels of resistance to propiconazole and flurprimidol compared with strain HRS10. Mutants with CYP65 and CYP68 overexpressed showed significantly reduced sensitivity to boscalid, and only the mutant with CYP561 overexpressed displayed resistance to iprodione (Fig. 3A). In addition, overexpression of *CYP561*, *CYP65*, and *CYP68* led to increases in the biotransformation rate of propiconazole. Especially, the biotransformation rate constants of propiconazole for 24 h in HRS10 with CYP561, CYP65, and CYP68 overexpressed were 2.6-, 3.1-, and 3.8-fold higher than those of propiconazole in HRS10, respectively (Fig. 3B). The biotransformation activities by three CYP450s might be due to catalyzation of propiconazole to produce hydroxylated (-OH) metabolites (21). Mutants with phase III transporter ShatrD and ShPDR1 overexpressed exhibited reduced sensitivities to chemically different fungicides and the plant growth regulator compared with strain HRS10 (Fig. 4A). Heterologous expression of *ShPDR1* or *ShatrD* in a drug-hypersensitive yeast mutant (AD12345678 [designated AD1–8 here]) further confirmed that these transporters are involved in MDR (Fig. 4B). Therefore, overexpression of three CYP450s and two ABC transporters contributes to multidrug resistance in the MDR strain by the phase I and III detoxification pathway.

### A xenobiotic detoxification regulator, ShXDR1, coordinately regulates phase I and III genes, and a gain-of-function mutation in ShXDR1 leads to MDR.

**FIG 5:**
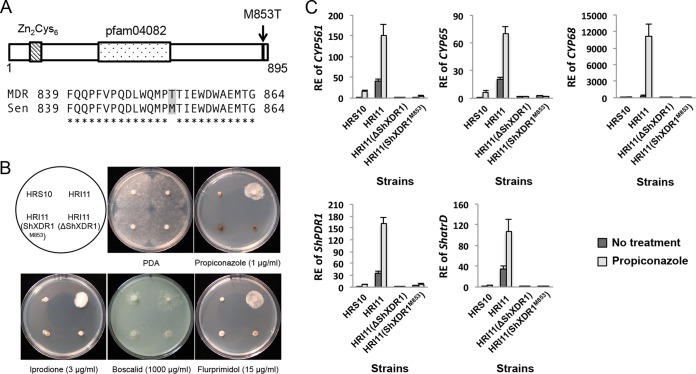
An activating mutation in a xenobiotic detoxification regulator 1 (ShXDR1) confers multidrug resistance by constitutive and induced overexpression of phase I and III genes. (A) Schematic diagram of the ShXDR1 transcription factor showing an amino acid substitution (methionine to threonine) in the MDR strains and alignment to the corresponding region of the mutation in sensitive (Sen) and MDR strains. (B) Sensitivity of strains HRS10, HRI11, HRI11(ΔShXDR1), and HRI11(ShXDR1^M853^) to fungicides and plant growth regulator. (C) Relative expression (RE) of phase I genes (*CYP561*, *CYP65*, and *CYP68*) and phase III genes (*ShPDR1* and *ShatrD*) before and after exposure to propiconazole (1 µg ml^−1^) for 40 min in strains HRS10, HRI11, HRI11(ΔShXDR1), and HRI11(ShXDR1^M853^).

To find the genetic factor leading to overexpression of three CYP450s and two ABC transporters in the MDR strain, we first compared the upstream regions of *CYP561* (1,314 bp), *CYP65* (843 bp), *CYP68* (645 bp), *ShPDR1* (1,566 bp) and *ShatrD* (1,654 bp) between strains HRS10 and HRI11 using genome sequences generated by Green et al. (22). The 459-bp upstream region of *ShPDR1* and 1,000-bp upstream region of *ShatrD* from strains HRS10 and HRI11 were reported in previous publications (19, 20). The comparison of these five genes’ upstream regions revealed that the regions of HRS10 and HRI11 are identical. Since the differential expressions of five genes between the strains HRS10 and HRI11 showed a similar pattern (Fig. 2A and B), we speculated that all of them might be simultaneously regulated and the MDR phenotype might be caused by a mutation(s) in one of the members of the fungus-specific Zn_2_Cys_6_ family, which have been known to be the regulator of ABC transporters in other fungal systems (3, 14, 23). We conducted a probability variant detection analysis between sequences of 78 putative Zn_2_Cys_6_ transcription factors from strains HRS10 and HRI11 and found that one of the transcripts (TCONS_00003992) has an amino acid substitution in the MDR strain HRI11 (Fig. 5A). The substitution is methionine (ATG) to threonine (ACG) at codon 853 (M853T) in xenobiotic detoxification regulator 1, ShXDR1, in strain HRI11. Sequencing of the transcription factor (ShXDR1) in additional five drug-sensitive and five MDR strains collected from the same site where HRI11 and HRS10 originated indicated that all five MDR strains contained this mutation, which was not found in the sensitive strains.

To confirm the *ShXDR1* gene encodes the transcription factor responsible for overexpression of five phase I and III genes, leading to multidrug resistance in MDR strain HRI11, we generated the ShXDR1 deletion mutants using a split marker approach and an approach with a CRISPR-Cas9 system, ShXDR1 knockdown mutants, and ShXDR1^M853^ mutants from strain HRI11. ShXDR1 deletion and knockdown and the replacement of ShXDR1^T853^ with ShXDR1^M853^ in strain HRI11 led to increased sensitivity to propiconazole, iprodione, boscalid, and flurprimidol (Fig. 5B; see Fig. S2A in the supplemental material) and decreased constitutive expression of five detoxification genes (Fig. 5C; Fig. S2B). Also, the expression of five genes was not induced by propiconazole in the ShXDR1 deletion mutant but induced by propiconazole in the HRI11(ShXDR1^M853^) mutant that showed a similar expression pattern to the sensitive strain HRS10 (Fig. 5C). In this study, an efficient target (ShXDR1) deletion method was developed using the split marker method with a CRISPR-Cas9 system in S. homoeocarpa. A propiconazole-sensitive mutant was not generated from protoplasts of HRI11 using polyethylene glycol (PEG)-mediated transformation with donor vector DNA only (Topo-ΔShXDR1), but the addition of two vectors containing Cas9 endonuclease and CRISPR RNA:transactivating CRISPR RNA (crRNA:tracrRNA) chimeric guide RNA with target ShXDR1 sequences, respectively, contributed to successful ShXDR1 deletions. Furthermore, the method with split marker DNA plus the CRISPR-Cas9 system displayed high success rates of ShXDR1 deletion mutants from HRI11, showing increased sensitivity to propiconazole and decreased expression of *ShatrD* (see Fig. S3 in the supplemental material).

10.1128/mBio.00457-18.3 FIG S2  *In vitro* chemical sensitivity and gene expression assay of ShXDR1 knockdown mutants from the multidrug-resistant strain HRI11. (A) Sensitivity of strains HRS10, HRI11, and HRI11(dsRNA-XDR1)-1 and -2 to fungicides and plant growth regulator. (B) Relative expression (RE) of phase I genes (*CYP561*, *CYP65*, and *CYP68*), phase III genes (*ShPDR1* and *ShatrD*), and transcription factor gene *ShXDR1* before and after exposure to propiconazole (1 µg ml^−1^) for 40 min in strains HRS10, HRI11, and HRI11(dsRNA-XDR1)-1 and -2. Download FIG S2, TIF file, 2 MB. Copyright © 2018 Sang et al. 2018 Sang et al. This content is distributed under the terms of the Creative Commons Attribution 4.0 International license.

10.1128/mBio.00457-18.4 FIG S3  An efficient target gene (*ShXDR1*) deletion using a CRISPR-Cas9 system. (A) The ratio of ShXDR1 deletion mutants in total hygromycin resistance transformants from the multidrug-resistant strain HRI11. The transformants were generated using four different combinations of constructs: donor only (Topo-ΔShXDR1), donor plus Cas9 (p426-SNR52p-gRNA.csr-1.Y-SUP4t) (50) plus gRNA-XDR1 (p426-SNR52p-gRNA.ShXDR1.Y-SUP4t), split marker approach only (one with 1 kb of upstream region of *ShXDR1* and 731 bp of hph and one with 1 kb of downstream region of *ShXDR1* and 1,126 bp of PtrpC-hph), and the split marker approach plus Cas9 plus gRNA-XDR1. Values in the bars followed by the same letter are not significantly different at *P* = 0.05. (B) Example of transformants (Trans 1 to Trans 8) displaying increased sensitivity to propiconazole. (C) Relative expression (RE) of *ShatrD* before and after exposure to propiconazole (1 µg ml^−1^) for 40 min in strain HRI11 and Trans 1 and Trans 2. Download FIG S3, TIF file, 1.8 MB. Copyright © 2018 Sang et al. 2018 Sang et al. This content is distributed under the terms of the Creative Commons Attribution 4.0 International license.

**FIG 6:**
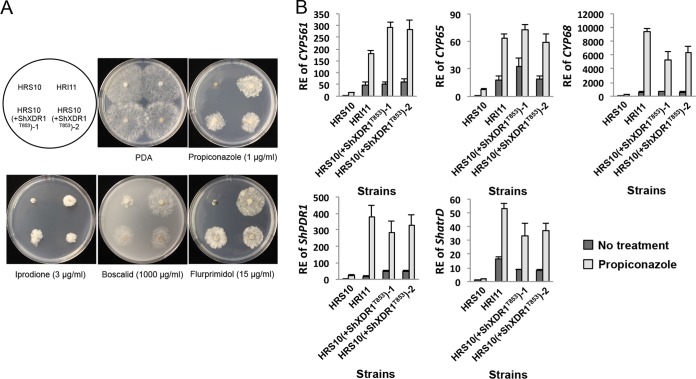
A mutation in a xenobiotic detoxification regulator 1 (ShXDR1) is a dominant gain-of-function mutation leading to multidrug resistance by constitutive and induced overexpression of phase I and III genes. (A) Sensitivity of strains HRS10, HRI11, and HRS10(+ShXDR1^T853^)-1 and -2 to fungicides and plant growth regulator. (B) Relative expression (RE) of phase I and III genes before and after exposure to propiconazole (1 µg ml^−1^) for 40 min in strains HRS10, HRI11, and HRS10(+ShXDR1^T853^)-1 and -2.

Finally, to test whether the M853T mutation is sufficient for the generation of the MDR phenotype in strain HRI11 and to determine whether this gain-of-function mutation is dominant or recessive, we transformed a plasmid with an upstream segment (1,581 bp) and the full length of ShXDR1^T853^ into strain HRS10. The mutant HRS10(+ShXDR1^T853^) exhibited multidrug resistance to different fungicides and the plant growth regulator, similar to strain HRI11 (Fig. 6A), and showed constitutive upregulation of the five detoxification genes (Fig. 6B). Taken together, these results indicated that the MDR phenotype in the strain HRI11 was caused by a dominant gain-of-function mutation, M853T, in ShXDR1.

### Interaction of ShXDR1 on promoter regions of phase I and III genes and additional ShXDR1 target gene regulon.

**FIG 7:**
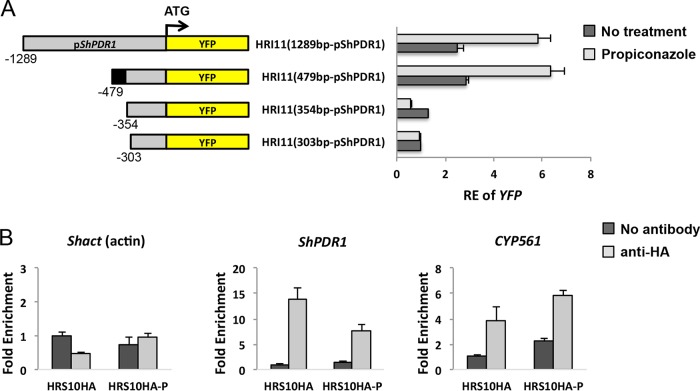
Detection of xenobiotic-responsive element (XRE) and interaction between ShXDR1 and the promoter region of the ShXDR1 target gene regulon. (A) Promoter deletion analysis of p*ShPDR1*-YFP chimeric constructs. The promoter 5′ deletions in *ShPDR1* are schematized with their corresponding plasmid constructs and S. homoeocarpa mutants. Relative expression (RE) of YFP is given for each mutant before and after exposure to propiconazole. The black region in −479 bp upstream of ShPDR1 indicates the potential promoter region containing the ShXDR1 binding motif. (B) Chromatin immunoprecipitation (ChIP) analysis of HA-tagged fusion ShXDR1 occupancy on promoters of *ShPDR1* (−448 bp to −342 bp) and *CYP561* (−493 bp to −387 bp) in the absence and presence of propiconazole. The presence of the promoter region of *ShPDR1*, *CYP561*, or *Shact* (as a negative control) sequence was assayed by quantitative PCR. The *y* axis depicts the fold enrichment over a mock immunoprecipitation control that lacks HA antibody. HRS10HA-P indicates HA-expressing strain HRS10 treated with propiconazole (1 µg ml^−1^).

To identify the ShXDR1 binding site in the promoter region of *ShPDR1*, 5′ deletions in each promoter were cloned in frame with the yellow fluorescent protein (YFP) reporter gene in pYHN3 (24), and the resulting constructs were transformed into strain HRI11. A 2.3-fold increase in relative expression of YFP was observed when the mutant HRI11-1289PDR1, containing the 1,289-bp upstream region of *ShPDR1* and the YFP chimeric construct, was exposed to propiconazole for 40 min. A deletion of the region between bp −1289 and −479 with respect to the ATG initiation codon had no effect on either constitutive YFP gene expression or propiconazole-induced YFP gene expression. Further deletion of the 5′ end of the promoter between bp −479 and −303 had a dramatic effect on the constitutive and propiconazole-induced YFP gene expression. This observation suggested the presence of a region containing an element participating in constitutive overexpression of *ShPDR1* due to ShXDR1 binding to this region and induced expression of *ShPDR1* by the propiconazole treatment (Fig. 7A). Chromatin immunoprecipitation (ChIP) experiments using hemagglutinin (HA) antibody directed against the DNA-binding domain of ShXDR1 confirmed that this protein was indeed bound to the promoter region of *ShPDR1* between bp −448 and −342 in strain HRS10 (Fig. 7B). The results from promoter deletion and ChIP analysis suggested the putative binding motif between bp −373 and −354 (5′ CGGCTGTTCAATAATACCG 3′) (underline indicates trinucleotide sequences might be recognized by zinc cluster proteins) on the promoter region of *ShPDR1*. Additional ChIP analysis indicated that ShXDR1 was also bound to the promoter region of *CYP561* between bp −493 and −387 (Fig. 7B), which contains the putative binding motif between bp −436 and −418 (5′ CGGATGTTACATTTACCG 3′). Using the MEME motif discovery tool, consensus inverted repeat sequences CGG(N12 or N13)CCG were found upstream of two other CYP450s and ShatrD, implying that ShXDR1 is bound to the predicted DNA binding motif in the promoter region of three CYP450s, ShPDR1, and ShatrD for the regulation of xenobiotic detoxification (see Fig. S4A in the supplemental material).

10.1128/mBio.00457-18.5 FIG S4  ShXDR1 target gene regulon. (A) Sequences and locations of putative binding motifs for ShXDR1 in the promoter regions of ShXDR1 target gene regulon. (B) Relative expression (RE) of the regulon with four additional ShXDR1 target genes before and after exposure to propiconazole (1 µg ml^−1^) for 40 min in strains HRS10, HRI11, HRI11(ΔShXDR1), and HRS10(+ShXDR1^M853^). Download FIG S4, TIF file, 0.4 MB. Copyright © 2018 Sang et al. 2018 Sang et al. This content is distributed under the terms of the Creative Commons Attribution 4.0 International license.

In addition to three CYP450 genes and two ABC transporter genes, four more genes from group A (Fig. 1A) were confirmed to be regulated by ShXDR1. The genes are involved with emopamil binding (TCONS_00010618), cellulose-binding family II (TCONS_0003057), lysophospholipase (TCONS_00002727), and hypothetical protein (TCONS_00023546). A BLASTX search revealed that the hypothetical protein shares low homology with 1-aminocyclopropane-1-carboxylate (ACC) deaminase from Streptomyces rubellomurinus (accession no. WP_045693734) and were identified by an E value of 0.78 and coding sequence coverage of 47%. The query sequence shares 30% of amino acid identity with ACC deaminase (32 out of 106). These four genes were constitutively overexpressed and induced by propiconazole in the MDR strain HRI11, but had decreased expression in the mutant HRI11(ΔShXDR1) and were overexpressed in HRS10(ShXDR1^T853^) (Fig. S4B). The consensus inverted repeat sequences CGG(N11 or N13)CCG was also found in the upstream region of these four genes using the MEME motif discovery tool (Fig. S4A).

### Conserved detoxification systems through transcriptional regulation of phase I and III genes in filamentous fungi.

**FIG 8:**
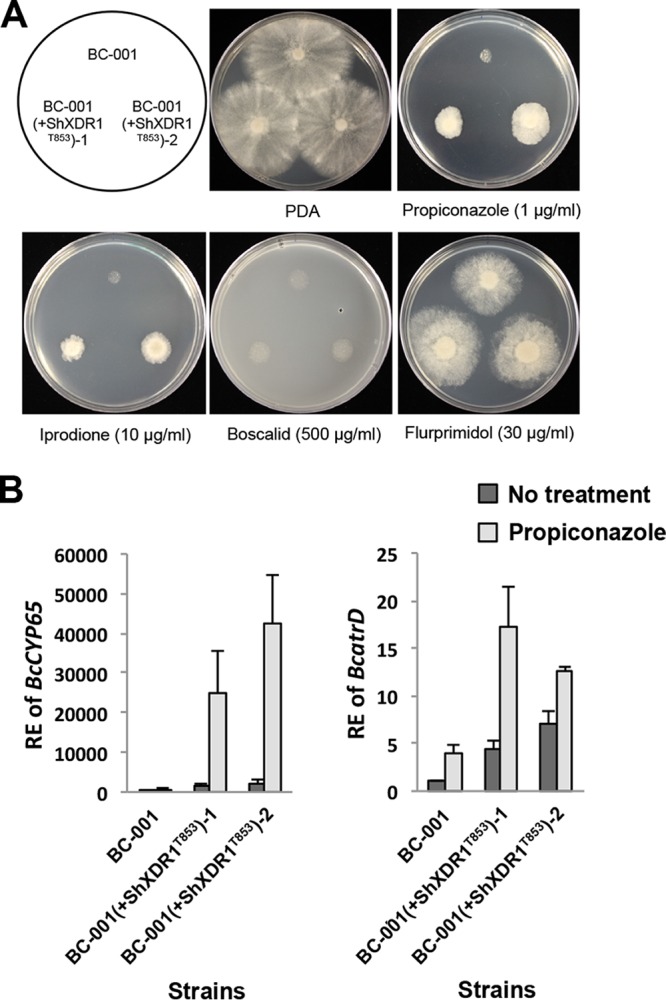
Conserved detoxification systems through transcriptional regulation of phase I and III genes in Botrytis cinerea. (A) Sensitivity of Botrytis cinerea strains BC-001 and BC-001(+ShXDR1^T853^)-1 and -2 to fungicides and plant growth regulator. (B) Relative expression (RE) of phase I gene *BcCYP65* and phase III gene *BcatrD* before and after exposure to propiconazole (1 µg ml^−1^) for 40 min in strains BC-001 and BC-001(+ShXDR1^T853^)-1 and -2.

To assess whether the xenobiotic detoxification pathway is conserved in other filamentous ascomycete fungi, we introduced ShXDR1^T853^ into a plant-pathogenic fungus, Botrytis cinerea. Two B. cinerea transformants containing ShXDR1^T853^ displayed increased resistance to propiconazole and iprodione, but not to boscalid and flurprimidol (Fig. 8A). The wild-type B. cinerea strain significantly induced expression of CYP450 enzyme gene *BcCYP65* and ABC transporter gene *BcatrD* in response to propiconazole. In the presence of ShXDR1^T853^, in B. cinerea, the *BcCYP65* and *BcatrD* genes were constitutively overexpressed and highly induced by propiconazole compared to the wild-type strain (Fig. 8B). The foreign transcription factor harboring the gain-of-function mutation confers the MDR phenotype in B. cinerea through regulation of phase I and III genes, indicating that the xenobiotic detoxification system is conserved in filamentous ascomycete fungi.

## DISCUSSION

Our systematic analysis of MDR in S. homoeocarpa using RNA-seq and genetic modification approaches has revealed key factors for xenobiotic detoxification. We have elucidated the molecular mechanisms of xenobiotic detoxification for the first time in fungi, which are through the coordinated transcriptional regulation of genes coding for phase I metabolizing enzymes, *CYP561*, *CYP65*, and *CYP68*, and phase III efflux transporters, *ShPDR1* and *ShatrD*, under the control of a fungus-specific transcription factor, *ShXDR1*. In addition, a gain-of-function mutation in ShXDR1 causes constitutive and drug-induced overexpression of phase I and III genes, contributing to MDR in the S. homoeocarpa field strain.

RNA-seq analysis of drug-sensitive and MDR strains indicated that constitutively overexpressed genes in the MDR strain were typically associated with xenobiotic detoxification (Fig. 1C; see Fig. S5A in the supplemental material). However, many of the constitutively overexpressed genes in the MDR strain were not induced by propiconazole, and only 13 transcripts (group A) including three CYPs and two ABC transporters, showed the propiconazole-induced expression pattern. These phase I and III genes were rapidly and transiently activated in response to structurally different chemicals. Overexpression of *CYP561*, *CYP65*, and *CYP68* conferred resistance to propiconazole and flurprimidol and displayed various insensitivities to boscalid and iprodione, which might be due to the different substrate specificity of CYPs. A BLAST search in the Fungal Cytochrome P450 Database (FCPD: http://p450.riceblast.snu.ac.kr) and phylogenetic analysis revealed that the products of *CYP561* and *CYP65* belong to clan CYP65 and the product of *CYP68* belongs to clan CYP68 (see Fig. S6A in the supplemental material). The functional classification of clans CYP65 and CYP68 was determined as secondary metabolism (for example, mycotoxin trichothecene biosynthesis by CYP65 and gibberellin biosynthesis by CYP68) (25, 26), but our findings suggest additional functions of clans CYP65 and CYP68 in xenobiotic metabolism. Screening of phase II enzymes from RNA-seq data revealed only one phase II glutathione *S*-transferase (TCONS_00005783) was highly overexpressed in the MDR strain, but the gene was not an ShXDR1 target gene regulon, and overexpression of the gene did not affect the biotransformation rate of propiconazole (Fig. S5B and C).

10.1128/mBio.00457-18.6 FIG S5  Phase II glutathione *S*-transferase. (A) Xenobiotic response and metabolism genes constitutively upregulated in Sclerotinia homoeocarpa multidrug-resistant (MDR) strain HRI11. (B) Relative expression (RE) of phase II glutathione *S*-transferase (TCONS_00005783) before and after exposure to propiconazole (1 µg ml^−1^) for 10, 20, 40, and 60 min in strains HRS10 and HRI11 and before and after exposure to propiconazole (1 µg ml^−1^) for 40 min in HRI11(ΔShXDR1). (C) Propiconazole biotransformation rate of strain HRS10 and the mutant with glutathione *S*-transferase (TCONS_00005783) overexpressed using HPLC analysis. Download FIG S5, TIF file, 0.4 MB. Copyright © 2018 Sang et al. 2018 Sang et al. This content is distributed under the terms of the Creative Commons Attribution 4.0 International license.

Mutants with the ABC transporter overexpressed exhibited broad-spectrum resistance to chemicals. Interestingly, *ShPDR1* conferred resistance to higher concentrations of chemicals compared to *ShatrD* in the heterologous expression system in yeast. In a mutagenesis study of ABC transporter Cdr1 transmembrane domains (TMDs) in C. albicans, only five mutations in transmembrane segments (TMSs) lead to reduced efflux and ATPase activity of Cdr1 (27). Three amino acids (F559, E564, and G672) in Cdr1 are conserved in Cdr2 from C. albicans and ShPDR1 and ShatrD, but one of the amino acids (T677) in TMS5 of Cdr1 matches with T693 in ShPDR1, but not Cdr2 and ShatrD (Fig. S6C). This amino acid difference might contribute to decreased efflux activity of Cdr2 or ShatrD to a variety of chemicals and a high concentration (28) because these 4 amino acids are associated with the open and closed conformations of the TMDs, affecting ATP hydrolysis by the nuclear binding domains (NBDs) (27). The phenotypes of S. homoeocarpa and S. cerevisiae mutants with ShPDR1 and ShatrD overexpressed in response to propiconazole also suggest that these transporters are involved in two steps (phase 0 and phase III) of disposal for parent xenobiotics and biotransformed xenobiotics, respectively, since propiconazole was biotransformed in S. homoeocarpa and not in S. cerevisiae (Fig. S6D). Our results propose that the activities of phase I genes *CYP561*, *CYP65*, and *CYP68* contribute to catalyzing xenobiotics with different substrate specificities, and phase III genes *ShPDR1* and *ShatrD* are involved in excretion of xenobiotics or their metabolites.

10.1128/mBio.00457-18.7 FIG S6  Phase I CYP450s and phase III transporters. (A) Neighbor-joining tree for amino acid sequences of clans CYP65 and CYP68 in fungi, animals, and plants with Sclerotinia homoeocarpa CYP561, CYP65, and CYP68. Asterisks indicate CYP450s known to be involved with biosynthesis of secondary metabolites. The scale bar represents the number of amino acid substitutions per site. Bootstrap values are for 1,000 replicates. (B) Sequence logos of the conserved CYP motifs from the fungi CYP561 and their comparison against human CYP561 (CYP3A43). (C) The alignment of amino acid sequences of transmembrane segments 2 and 5 (TMS 2 and 5) in ABC transporters ShatrD and ShPDR1 and Candida albicans CaCDR1 and CaCDR2. Asterisks indicate identical amino acids, and colons indicate similar amino acids among the transporters compared. (D) Propiconazole biotransformation rate of strain AD1–8-pYES2 containing empty vector and AD1–8:PDR1-1 containing the ShPDR1 expression vector using HPLC analysis. Download FIG S6, TIF file, 1.9 MB. Copyright © 2018 Sang et al. 2018 Sang et al. This content is distributed under the terms of the Creative Commons Attribution 4.0 International license.

We found the xenobiotic detoxification regulator ShXDR1 has two highly conserved domains in comparison with Pdr1 from S. cerevisiae and Tac1 from C. albicans (see Fig. S7A in the supplemental material). The first conserved domain is a cysteine-rich motif at the N terminus, involved in zinc-dependent binding to DNA, and the other domain is a part of xenobiotic binding domain (XDB) in the middle of the protein (3). The ChIP and promoter deletion analysis suggests that this DNA binding domain (DBD) of ShXDR1 constitutively binds to the upstream region of *ShPDR1* and *CYP561*, and likely others, and possibly binds as a homodimer to its DNA binding motif, CGG(N12–13)CCG. The presence of the XDB suggests that activated ShXDR1 may initiate transcriptional expression of phase I and III genes through direct binding of xenobiotics. In addition to xenobiotic binding, the substrates of ShXDR1 could be endobiotics because *CYP65*, *CYP68*, *ShPDR1*, and *ShatrD* were constitutively overexpressed without treatment with any chemicals in the HRS10 mutant with CYP561 overexpressed (see Fig. S8 in the supplemental material). Phylogenetic analysis and MDR phenotypes of the B. cinerea strain caused by ShXDR1^T853^ expression reveal that ShXDR1 orthologs are conserved in genomes of filamentous ascomycete fungi capable of xenobiotic detoxification (Fig. 8; Fig. S7B). Thakur et al. provided evidence of mechanistic similarities for MDR regulation between fungal zinc cluster Pdr1 orthologs and the mammalian PXR nuclear receptor (3), but Pdr1 orthologs do not regulate the expression of phase I enzymes, which are a main factor of PXR regulation for xenobiotic detoxification. This absence of phase I regulation in Pdr1 might be attributed to extensive CYP450 gene loss in ascomycete yeasts (29), and this fungal group contains small CYP-omes lacking some globally or locally conserved CYP families, including clans CYP65 and CYP68 (25, 29), whereas, filamentous ascomycete fungi, including S. homoeocarpa, comprise the large number of members of the CYP family/subfamilies (29, 30). The coordinated regulation of CYPs and ABC transporters by ShXDR1 indicates that the fungal transcription factor is mechanistically analogous to PXR, which regulates CYP450s (especially CYP3A), and the P-glycoprotein family of ABC transporters (31, 32). Intriguingly, the fungal CYP561 family in clan CYP65 is phylogenetically close to two human CYP3A43 proteins identified as belonging to the CYP561 family, and both groups of proteins share the amino acid sequences in conserved protein domain family CypX (COG2124) (Fig. S6B). In addition, both S. homoeocarpa CYPs and human CYP3A enzymes have the ability to detoxify propiconazole (20), and upregulation of CYPs and ABC transporters by ShXDR1/PXR is a main factor for multidrug resistance in S. homoeocarpa and human cancer (9, 33, 34). This evidence proposes that the set of three components—CYPs, ABC transporters, and ShXDR1/PXR—is a key factor for xenobiotic detoxification in eukaryotes. Importantly, these evolutionarily parallel fungal and mammalian CYPs and ABC transporters regulated by the structurally unrelated transcription factors ShXDR1 and PXR, suggesting the detoxification system in eukaryotes has evolved convergently.

10.1128/mBio.00457-18.8 FIG S7  Alignment of the DNA binding domain and xenobiotic binding domain from ShXDR1 and other known transcription factors and phylogenetic analysis of ShXDR1 homologues in ascomycete fungi. (A) Alignment of amino acid sequences of the DNA binding domain and xenobiotic binding domain in ShXDR1, Candida albicans Tac1, and Saccharomyces cerevisiae Pdr1. Asterisks indicate identical amino acids, and colons indicate similar amino acids among the transcription factors compared. (B) Neighbor-joining tree for amino acid sequences of ShXDR1 homologues and other fungus-specific transcription factors known to be involved with multidrug resistance (*). The scale bar represents the number of amino acid substitutions per site. Bootstrap values are for 1,000 replicates. Download FIG S7, TIF file, 0.4 MB. Copyright © 2018 Sang et al. 2018 Sang et al. This content is distributed under the terms of the Creative Commons Attribution 4.0 International license.

10.1128/mBio.00457-18.9 FIG S8  Relative expression (RE) of phase I genes (*CYP561*, *CYP65*, and *CYP68*) and phase III genes (*ShPDR1* and *ShatrD*) before and after exposure to propiconazole (1 µg ml^−1^) for 40 min in strains HRS10 and HRS10(CYP561^OX^). Download FIG S8, TIF file, 0.2 MB. Copyright © 2018 Sang et al. 2018 Sang et al. This content is distributed under the terms of the Creative Commons Attribution 4.0 International license.

In this study, we determined a dominant gain-of-function mutation (M853T) in the activation domain of ShXDR1 renders constitutive overexpression of phase I and III genes responsible for multidrug resistance. C. albicans azole-resistant strains also contain a gain-of-function mutation in the activation domain of Tac1 (N972D, N997D, and G980E) that confers constitutive upregulation of the ABC transporter genes *CDR1* and *CDR2*, resulting in increased antifungal resistance (10, 12, 35). In addition, an activation mutation (F815S) in Pdr1 of S. cerevisiae enhances occupancy of coactivator complexes at the ABC transporter PDR5 promoter accompanied by loss of contacts between histones and DNA, and it also alters chromatin structure at both promoter and coding sequences of PDR5 (36). Since the mutation in ShXDR1 occurs in the C-terminal region that harbors the transcriptional activation domain, the mutation might affect the interaction between ShXDR1 and a coactivator or repressor. The activation domain of S. cerevisiae Pdr1 interacts with the KIX domain of the gal11 mediator coactivator, which recruits RNA polymerase II for transcription of ABC transporters (3). We identified a putative coactivator, gal11 (TCONS_00010521), containing a gal11 coactivator domain (accession no. cd12191), but gal11 was not a coactivator of ShXDR1, which was confirmed by gal11 deletion mutants in the MDR strain. Further studies will mine the candidate coactivators that potentially interact with ShXDR1 and compare the interaction of the coactivator between ShXDR1^M853^ and ShXDR1^T853^.

Additional parts of the ShXDR1 regulon are four genes encoding enopamil binding, phospholipase B1, cellulose-binding family II, and a hypothetical protein. Emopamil binding proteins (EBPs) are integral membrane proteins of the endoplasmic reticulum and bind sigma ligands and structurally diverse drugs and fungicides on the emopamil binding domain (EBD) (37). The protein may contribute to phase III of the xenobiotic detoxification system because EBPs share similar structural features with drug transporters, especially due to a high content of aromatic amino acid residues in transmembrane segments (38), which have been suggested to be involved in the drug transport by the P-glycoprotein (39). In addition, both phospholipase B1 (Plb1) and cellulose-binding family II are enzymes involved in hydrolysis of glycerophospholipids and cellulose, respectively (40, 41). Although the phase I reaction may occur by hydrolysis, the involvement of Plb1 and cellulose-binding family II in xenobiotic detoxification is unknown.

Our first establishment of a xenobiotic detoxification mechanism through metabolizing enzymes and efflux transporters coordinately regulated by a transcription factor in fungi may aid in the discovery of new target genes for fungicides to control MDR populations in plant and human filamentous pathogenic fungi. For example, the detailed molecular knowledge of the MDR mechanisms in C. glabrata facilitated the discovery of a small-molecule compound, iKIX1, that inhibits the interaction of the Pdr1 activation domain with the Gal11A KIX domain for resensitizing drug-resistant C. glabrata to azole antifungals (3, 13). Furthermore, the results describing a mutation in the regulator from this study will be used to understand a protein conformational flexibility to posit an explanation for ShXDR1 substrate promiscuity. Also, development of a detection tool for fungicide resistance populations will be helpful for better management of dollar spot on golf courses. The mechanistic similarities of ShXDR1 and PXR regulation provide the impetus for further exploration of PXR-mediated MDR in human cancer using the fungal system and further studies of convergent and divergent evolution of xenobiotic detoxification in eukaryotes.

## MATERIALS AND METHODS

### Fungal strains.

Fungal strains used in this study are presented in Table 1. Ten strains of S. homoeocarpa used in this study were collected from Hickory Ridge Golf club (Amherst, MA) and were previously characterized in field efficacy, fungicide sensitivity, and fungicide resistance molecular mechanism studies (19, 20, 42). Strains HRS10 and HRI11 were sampled prior to treatment of a demethylation inhibitor fungicide (propiconazole). Strains HRS1 to HRS4 were sampled from plots that received no fungicide treatment, and HRI1 to HRI4 were sampled 7 days after treatment. (Strains HRI1 to -4 were considered to demonstrate “practical field resistance.”) HRS10 and HRS1 to HRS4 were considered fungicide-sensitive strains based on a previous *in vitro* fungicide sensitivity assay. HRI11 and HRI1 to HRI4 are considered multidrug-resistant (MDR) strains and exhibited reduced sensitivities to propiconazole, iprodione, and boscalid (19).

**TABLE 1:** Fungal strains used in this study

Strain	Description	Source
HRS10	S. homoeocarpa drug-sensitive strain	Sang et al. (19)
HRI11	S. homoeocarpa MDR strain	Sang et al. (19)
HRS1 to -4	S. homoeocarpa drug-sensitive strains	Sang et al. (19)
HRI1 to -4	S. homoeocarpa practical field resistance strains	Sang et al. (19)
HRS10(CYP561^OX^)	HRS10 mutant with CYP561 overexpressed	This study
HRS10(CYP65^OX^)	HRS10 mutant with CYP65 overexpressed	This study
HRS10(CYP68^OX^)	HRS10 mutant with CY68 overexpressed	This study
HRS10(ShPDR1^OX^)	HRS10 mutant with ShPDR1 overexpressed	This study
HRS10(ShatrD^OX^)	HRS10 mutant with ShatrD overexpressed	This study
HRS10(+ShXDR^T853^)-1 and -2	HRS10 mutants with ShXDR1^T853^ expressed	This study
HRI11(∆ShXDR1)	HRI11 ShXDR1 knockout mutant	This study
HRI11(∆ShXDR1 + ShXDR1^T853^)-1 and -2	HRI11(ΔShXDR1) complemented mutants	This study
HRI11(ShXDR1^M853^)	HRI11 mutant with ShXDR1^M853^ expressed	This study
HRI11(ShXDR1^M853^ + ShXDR1^T853^)-1 and -2	HRI11(ShXDR1^M853^) complemented mutants	This study
HRI11(303bp-pShPDR1)	HRI11 mutant with 303-bp upstream region of ShPDR1-YFP chimeric construct	This study
HRI11(354bp-pShPDR1)	HRI11 mutant with 354-bp upstream region of ShPDR1-YFP chimeric construct	This study
HRI11(479bp-pShPDR1)	HRI11 mutant with 479-bp upstream region of ShPDR1-YFP chimeric construct	This study
HRI11(1289bp-pShPDR1)	HRI11 mutant with 1,289-bp upstream region of ShPDR1-YFP chimeric construct	This study
HRI11(dsRNA-ShXDR1)-1 and -2	HRI11 ShXDR1 knockdown mutants	This study
HRS10HA	HRS10 mutant with HA-tagged ShXDR1	This study
HRS10(TNCOS_00005783)	HRS10 mutant with glutathione *S*-transferase overexpressed	This study
BC-001	Botrytis cinerea strain	This study
BC-001(+ShXDR1^T853^)-1 and -2	BC-001 mutants with ShXDR1 expressed	This study
AD12345678 (AD1–8)	Saccharomyces cerevisiae drug-hypersensitive mutant	Decottignies et al.^a^
AD1–8-pYES2	AD1–8 mutant with empty pYES2 vector	Sang et al. (19)
AD1–8:PDR-1 and -2	AD1–8 mutant with ShPDR1 overexpressed	Sang et al. (19)
AD1–8:atrD-1 and -2	AD1–8 mutant with ShatrD overexpressed	This study

a See Text S1 in the supplemental material.

10.1128/mBio.00457-18.1 TEXT S1  Supplemental materials and methods. Download TEXT S1, PDF file, 1.6 MB. Copyright © 2018 Sang et al. 2018 Sang et al. This content is distributed under the terms of the Creative Commons Attribution 4.0 International license.

### RNA sequencing and transcript abundance.

An Illumina Hiseq 2000 was used to sequence two biological replicates under each condition, yielding over 75 million Hiseq 2000 reads for every condition. Hisat2 version 2.0.4 was used to create the alignment files onto the genome of HRI11 (accession no. LNKV00000000). Predicted transcripts of the recently sequenced Sclerotinia homoeocarpa genomes (22) were used to guide transcript assembly by Cufflinks using the –g flag. Transcript abundance was estimated by fragments per kilobase of transcript per million fragments of mapped reads (FPKM). A total of 12,008 genes were identified from the 28,914 transcripts. Significantly expressed genes were identified by log_2_ fold change greater than 1.5 and a *P* value below 0.05. Transcripts with FPKM below a 0.05 threshold were discarded in the differential expression analysis. Cluster analysis and heat map generation were conducted with the Genesis software (version 1.7.7) based on the hierarchical and complete linkage clustering method (http://genome.tugraz.at) (43). For functional annotation, the Blast2GO analysis was performed at https://www.blast2go.com. The predicted S. homoeocarpa coding sequences were searched for identical sequences by conducting BLASTX search. A cutoff E value of ≤10^−10^ was used for BLASTX and annotation.

### Plasmid construction and generation of S. homoeocarpa and B. cinerea mutants.

For overexpression of phase I genes (*CYP561*, CYP65, and *CYP68*) and phase III genes (*ShPDR1* and *ShatrD*), plasmid pYHN3-ptrpC was constructed by cutting pShEF1α and inserting ptrpC in plasmid pYHN3-pShEF1α (44). The full length of five genes amplified from genomic DNA (gDNA) of HRI11 was inserted into plasmid pYHN3-ptrpC to generate the plasmids pYHN3-ptrpC-CYP561, -CYP65, -CYP68, -ShPDR1, and -ShatrD, respectively. Each plasmid DNA (5 µg) was used for a polyethylene glycol (PEG)-mediated transformation in protoplasts from HRS10. The protoplast generation and PEG-mediated transformation were conducted according to Sang et al. (44). Phase I and III overexpression mutants HRS10(CYP561^OX^), HRS10(CYP65^OX^), HRS10(CYP68^OX^), HRS10(ShPDR1^OX^), and HRS10(ShatrD^OX^) were confirmed by quantitative PCR (qPCR) analysis, and the genes in those mutants were expressed 2,190-, 277-, 97,024-, 68-, and 13-fold more than in HRS10, respectively.

The transcription factor (TF) ShXDR1^T853^ containing mutation M853T is from MDR strain HRI11, and the wild-type TF, ShXDR1^M853^, is from HRS10. The HRI11 ShXDR1 deletion and ShXDR1^M853^ mutants were generated using the split marker approach and PEG-mediated protoplast transformation as described earlier (44). For deletion of *ShXDR1*, a 1-kb upstream region and downstream region of the *ShXDR1* amplified from genomic DNA of HRI11 were inserted into between the hygromycin resistance cassette (PtrpC-hph) in the plasmid Topo-hph (44) to generate the plasmid Topo-ΔShXDR1. Two constructs (one with 1 kb of upstream region of the *ShXDR1* and 731 bp of hph and one with 1 kb of downstream region of the *ShXDR1* and 1,126 bp of PtrpC-hph) were amplified from plasmid Topo-ΔShXDR1. For ShXDR1^M853^ mutants, Topo-ShXDR1^M853^ was constructed by cutting the upstream region of ShXDR1 in Topo-ΔShXDR1 and inserting the partial region of ShXDR1^M853^ from HRS10. Two constructs (one with the partial region of the *ShXDR1* and 731 bp of hph and one with 1 kb of downstream region of the *ShXDR1* and 1,126 bp of PtrpC-hph) were amplified from plasmid Topo-ShXDR1^M853^. The purified two constructs (each 2 µg) were used for the PEG-mediated transformation in protoplasts from HRI11. Mutants HRI11(ΔShXDR1) and HRI11(ShXDR1^M853^) were confirmed by PCR amplification with four primer pairs according to the method described by Sang et al. (44). ShXDR1^M853^ in mutant HRI11(ShXDR1^M853^) was sequenced to confirm the replacement. For the complementation of the mutants, the plasmid NeoR-ShXDR1^T853^ containing the neomycin resistance gene and upstream region (1,581 bp) and the full length of ShXDR1^T853^ was transformed into protoplasts of HRI11(ΔShXDR1) and HRI11(ShXDR1^M853^), respectively. Complemented mutants gained resistance to propiconazole and validated the phenotypes of the mutants HRI11(ΔShXDR1) and HRI11(ShXDR1^M853^). For ShXDR1 mutants from HRS10 and B. cinerea strain BC-001, the 1,581 bp of upstream region and full length of ShXDR1^T853^ amplified from gDNA of HRI11 were inserted into plasmid pYHN3-MCS (44) to generate plasmid pYHN3-ShXDR1^T853^. The plasmid DNA (5 µg) was used for the PEG-mediated transformation in protoplasts from HRS10 and BC-001. The presence of ShXDR1^T853^ transcript in BC-001(ShXDR1^T853^) mutants was confirmed by qPCR analysis.

pShPDR1-YFP chimeric mutants were generated for promoter deletion analysis. Different lengths (303, 354, 479, and 1,289 bp) of ShPDR1 promoter were amplified from gDNA of HRI11 and inserted into plasmid pYHN3 (24) to generate pYHN3-303bp-pShPDR1, pYHN3-354bp-pShPDR1, pYHN3-479bp-pShPDR1, and pYHN3-1289bp-pShPDR1. Five micrograms of each plasmid was transformed into protoplasts from HRI11. In addition, the HA-tagged fusion ShXDR1 mutant was generated for chromatin immunoprecipitation. The full length of ShXDR1^M853^ without a stop codon (TAA) from gDNA of HRS10 was fused with HA tag from plasmid pPAD80 (45) by two rounds of PCR. The HA-tagged fusion ShXDR1 was inserted into plasmid pYHN3-ptrpC to generate plasmid pYHN3-ptrpC-ShXDR1HA. This plasmid (5 µg) was used for PEG-mediated transformation in protoplasts from HRS10. The presence and overexpression of ShXDR1HA transcript in HRS10HA mutant were confirmed by qPCR analysis.

### HPLC analysis.

Potato dextrose broth (PDB; 50 ml) with and without mycelia (approximately 1 g) from strain HRS10 and mutants with CYP561, CYP65, and CYP68 overexpressed was supplemented with propiconazole (1 µg ml^−1^) and cultured at 25°C (100 rpm). The sample from extracellular growth medium was prepared at each time point (0, 6, 12, 24, 48, and 72 h) by a methanol extraction method (46). An Agilent 1200 Series high-performance liquid chromatography (HPLC) equipped with a diode array detector (DAD) was used for the detection and quantification of propiconazole. Separation was performed on an Agilent Eclipse XDB C_18_ column (4.6 by 150 mm, 5 µm) using a 12-min linear gradient of acetonitrile in water (50 to 90%) at a flow rate of 1.5 ml min^−1^. The DAD was set at 220 nm and the UV spectra from 190 to 400 nm were recorded for detection of transformation products.

### Quantitative real-time RT-PCR.

Total RNA of S. homoeocarpa was extracted using the methods detailed by Sang et al. (19). cDNA synthesis from total RNA was conducted with the QuantiTect reverse transcription kit (Qiagen, USA). The cDNA was used at an 8-fold dilution, and 1 µl was used for real-time quantitative PCR with Maxima SYBR green (Thermo Fisher Scientific, USA). The S. homoeocarpa actin gene (*Shact*) was selected as a housekeeping gene, and the primers for *ShatrD*, *ShPDR1*, and *Shact* were described by Hulvey et al. (20) and Sang et al. (19). Other primers used in qPCR were described in Table S1 in the supplemental material. The comparative threshold cycle (*C*_*T*_) method was used for calculation of relative gene expression (47).

10.1128/mBio.00457-18.10 TABLE S1  Plasmids and primers used in this study. Download TABLE S1, PDF file, 1.7 MB. Copyright © 2018 Sang et al. 2018 Sang et al. This content is distributed under the terms of the Creative Commons Attribution 4.0 International license.

### Chemical sensitivity assays.

*In vitro* sensitivity assays were conducted on the S. homoeocarpa strains with different classes of fungicides and plant growth regulator. Flurprimidol EC_50_ values for all 10 field strains were obtained using the method from Sang et al. (19). The concentrations of flurprimidol used for measuring the EC_50_ were 0.1, 1, 10, 100, and 1,000 µg ml^−1^. The propiconazole and iprodione EC_50_ values and boscalid EC_95_ values from 10 field strains previously used by Sang et al. (19) were reanalyzed in the present study. For *in vitro* sensitivity tests of S. homoeocarpa mutants and the control strains (HRS10 and HRI11), all strains were grown for 3 days at room temperature, and agar plugs (5 mm in diameter) were extracted from active colonies and inoculated onto the center of nonamended potato-dextrose agar (PDA) plates and each of the chemical-amended PDA plates. After 2 to 5 days, pictures of plates were taken (10 days for HRS10 mutants with ShPDR1 and ShatrD overexpressed on PDA amended with flurprimidol), and two diameters of the colony were measured using 16EX digital calipers (Mahr, Göttingen, Germany). The relative mycelium growth (RMG) percentages of strains were calculated. The experiment was repeated three times.

### Chromatin immunoprecipitation.

The procedure for chromatin immunoprecipitation was modified from Boedi et al. (48). Briefly, S. homoeocarpa mycelia grown in PDB (Becton, Dickinson, USA) for 4 days were not treated or were treated with 1 µg/ml of propiconazole for 40 min. The mycelia were fixed with 1% of formaldehyde for 15 min and incubated with glycine (125 mM) for 5 min using a shaker at 50 rpm. The mycelia were recovered by filtration, rinsed twice with 20 ml phosphate-buffered saline (PBS), and frozen in liquid nitrogen. The mycelia were ground to fine power with a mortar and pestle in liquid nitrogen, and 100 mg of ground mycelia was suspended in 1.5 ml of nucleus lysis buffer (50 mM HEPES-KOH, pH 7.5, 140 mM NaCl, 1 mM EDTA, 1% Triton X-100, 0.1% sodium deoxycholate, 1 mM phenylmethylsulfonyl fluoride [PMSF], with 1× protease inhibitor from Sigma). After the sample was centrifuged at 12,000 × *g* at 4°C for 10 min, the chromatin (supernatant) was transferred to a new tube. The chromatin was subjected to sonication for 25 s 12 times at power setting 35% with a Sonics VibraCell sonicator (Sonics & Materials, USA) and clarified by centrifugation at 12,000 × *g* at 4°C for 5 min. Six hundred microliters of chromatin was incubated with prewashed protein A magnetic beads (Thermo Scientific, USA) at 4°C for 1 h, and chromatin was separated from the beads using a magnetic stand. Prewashed anti-HA magnetic beads (Thermo Scientific, United States) were added to chromatin, and the mixture was incubated at 4°C overnight for immunoprecipitation. The bead-bound material was recovered using a magnetic stand, washed twice in 1 ml of low-salt washing buffer (20 mM Tris-HCl, pH 8.0, 150 mM NaCl, 2 mM EDTA, 0.5% Triton X-100, 0.1% SDS), twice in 1 ml of high-salt washing buffer (20 mM Tris-HCl, pH 8.0, 500 mM NaCl, 2 mM EDTA, 0.5% Triton X-100, 0.1% SDS), twice in 1 ml of LiCl washing buffer (10 mM Tris-HCl, pH 8.0, 0.5% NP-40, 1 mM EDTA, 0.5% sodium deoxycholate, 0.25 M LiCl·H_2_O), and twice in 1 ml of Tris-EDTA (TE: 10 mM Tris-HCl, pH 8.0, 1 mM EDTA). Beads were then resuspended in elution buffer (1% SDS, 0.1 M NaHCO_3_) and incubated with NaCl (5 M) at 65°C overnight to reverse cross-links. After the cross-links were reversed, 0.5 M EDTA, 1 M Tris-HCl (pH 6.5), and 10 mg ml^−1^ proteinase K were added to the sample, and the mixture was incubated at 45°C for 1 h. DNA from the sample was extracted with chloroform, precipitated with ethanol, 0.3 M sodium acetate (pH 5.2), and 1 µl glycogen (20 mg/ml), and resuspended in 50 µl double-distilled H_2_O (ddH_2_O). The purified DNA was analyzed by qPCR.

### Statistical significance tests.

All statistical analyses were performed with the JMP software package, version 10.0 (SAS Institute, Inc.).

### Accession number(s).

Reads generated in this study have been deposited in the National Center for Biotechnology Information Sequence Read Archive under accession no. SRP116271.
