# Supplementary figures and images for: A Xenobiotic Detoxification Pathway through Transcriptional Regulation in Filamentous Fungi
Source: mBio. 2018 Jul 17;9(4):e00457-18. doi: 10.1128/mBio.00457-18 (PMC6050962; doi:10.1128/mBio.00457-18)

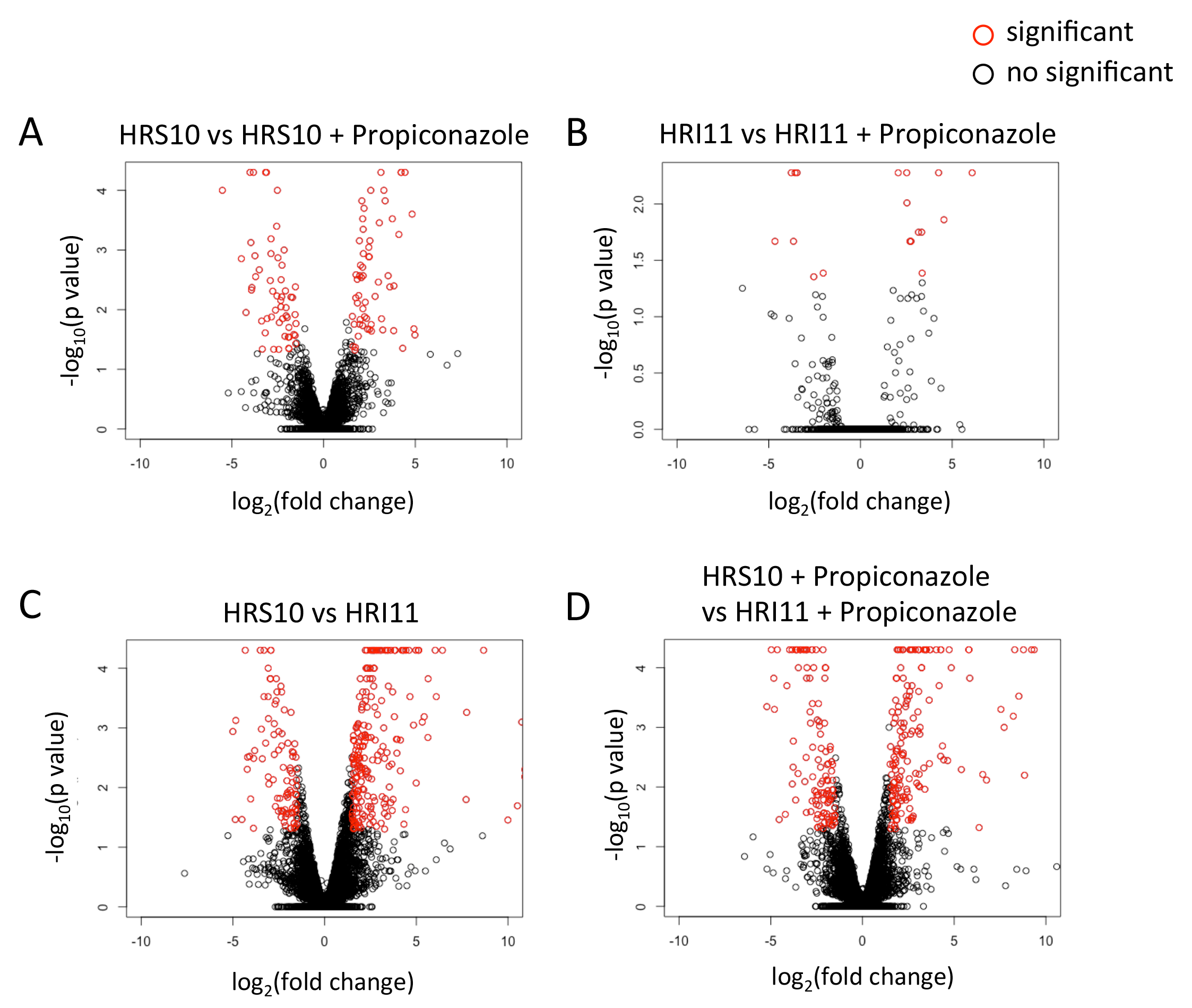

Supplement: FIG S1 [file mbo004183984sf1.tif]

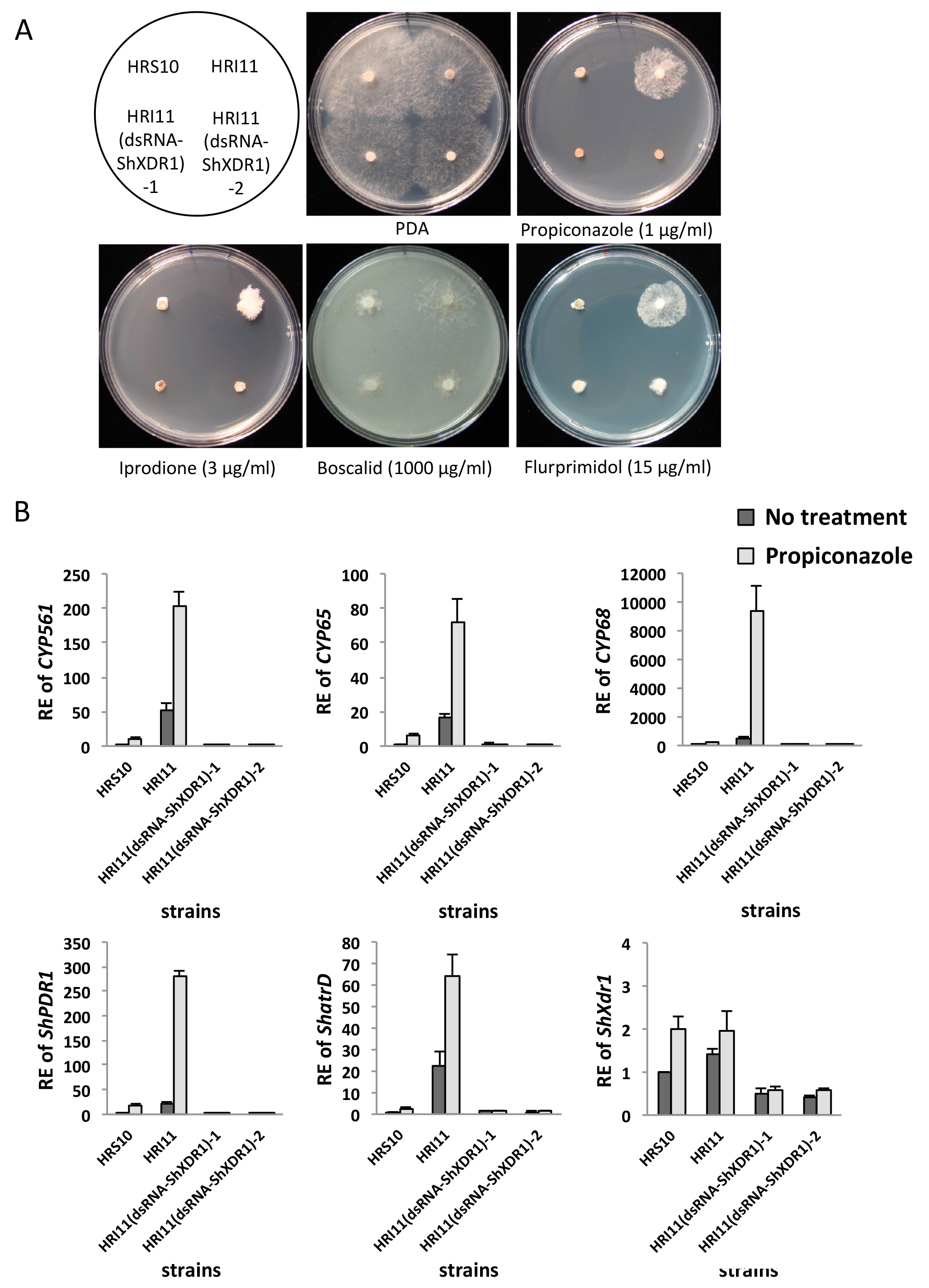

Supplement: FIG S2 [file mbo004183984sf2.tif]

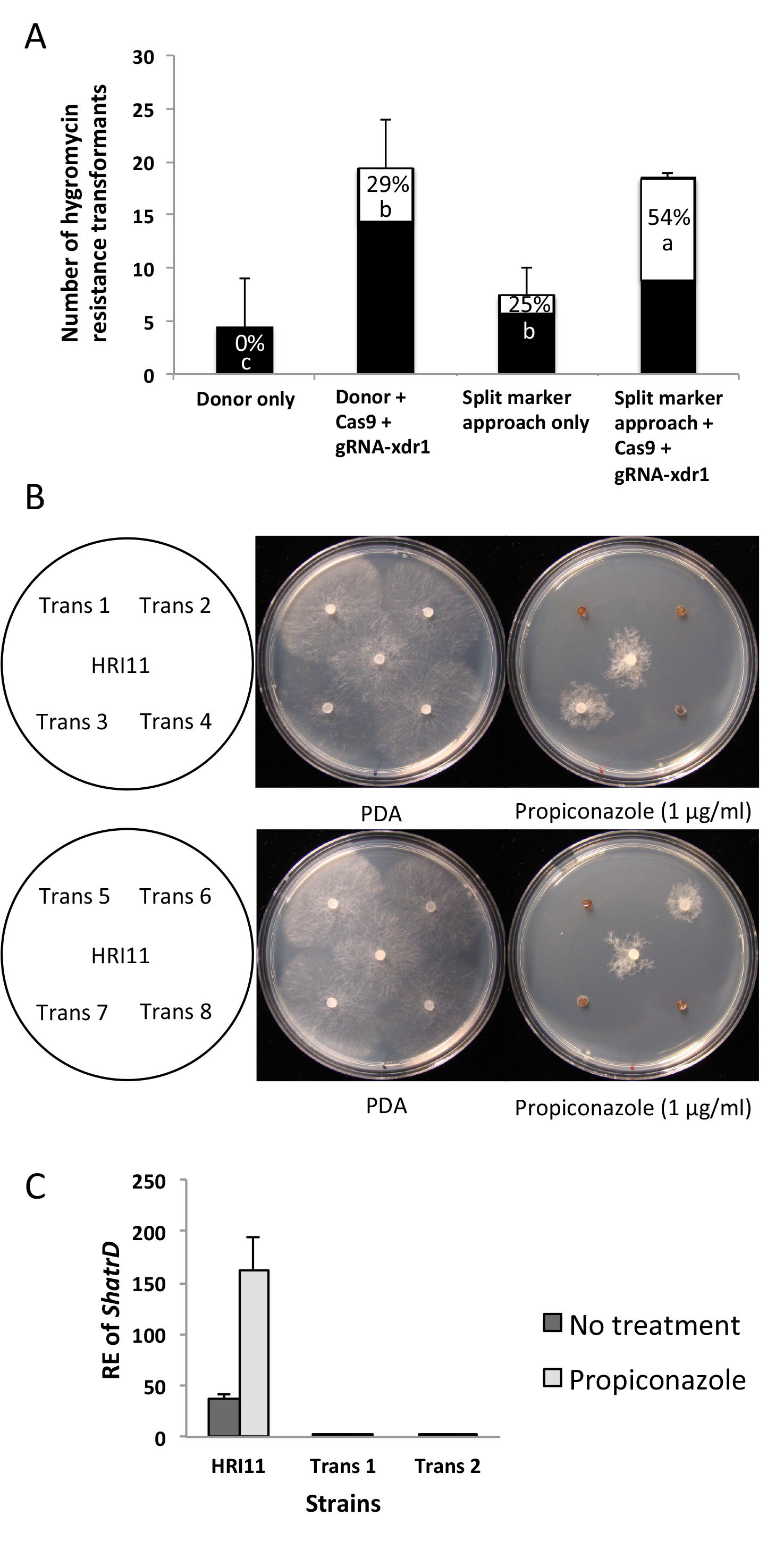

Supplement: FIG S3 [file mbo004183984sf3.tif]

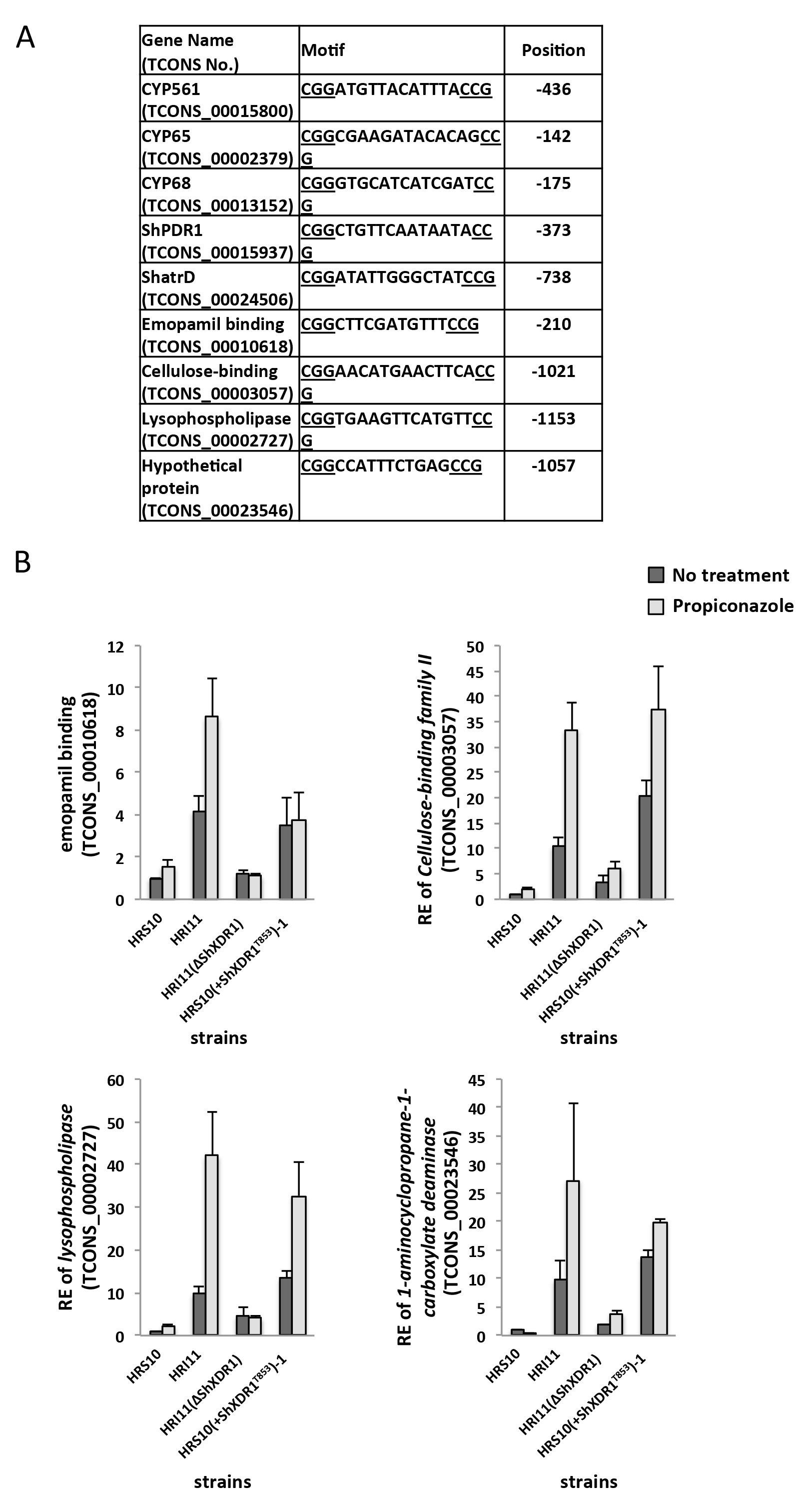

Supplement: FIG S4 [file mbo004183984sf4.tif]

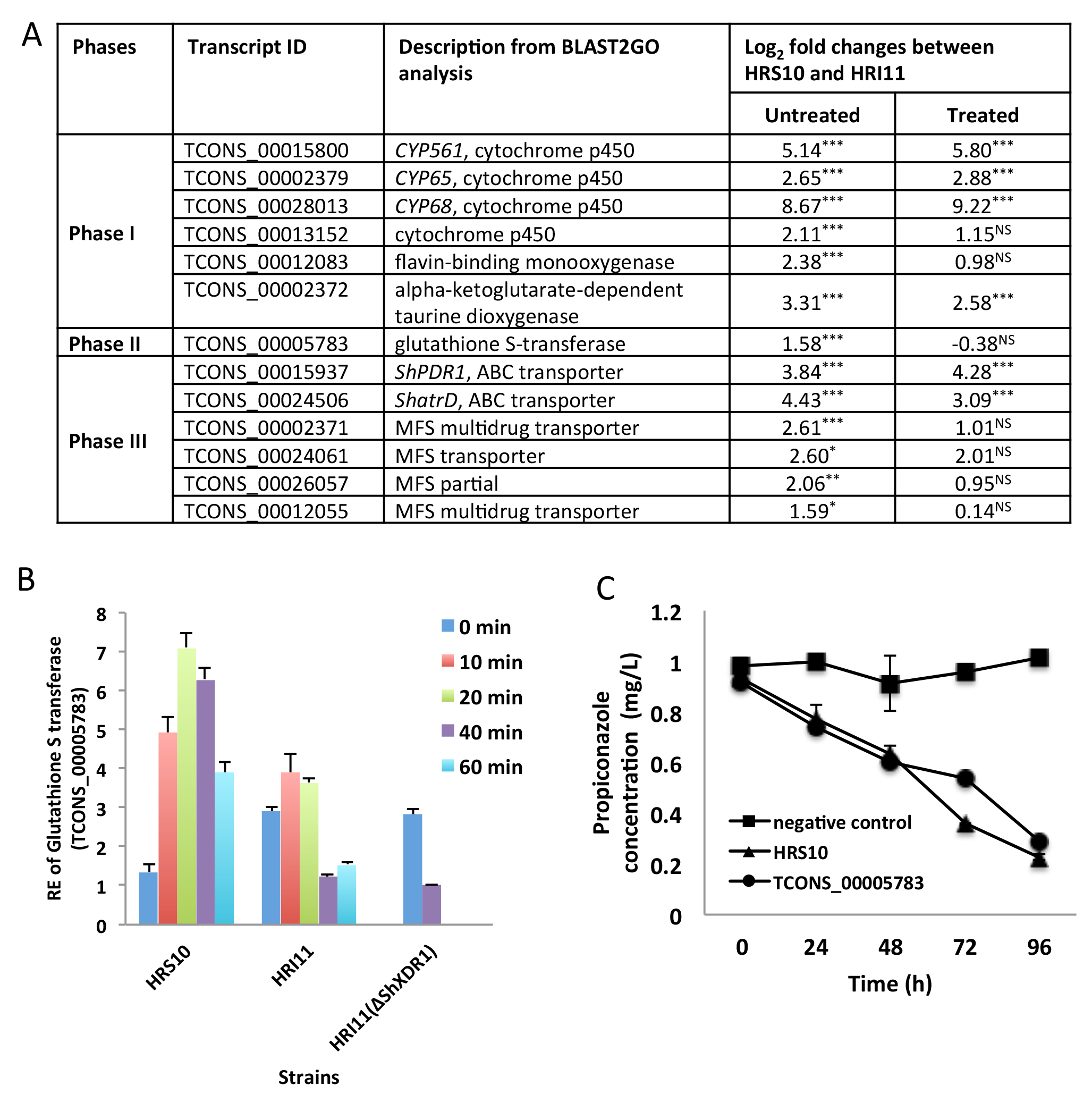

Supplement: FIG S5 [file mbo004183984sf5.tif]

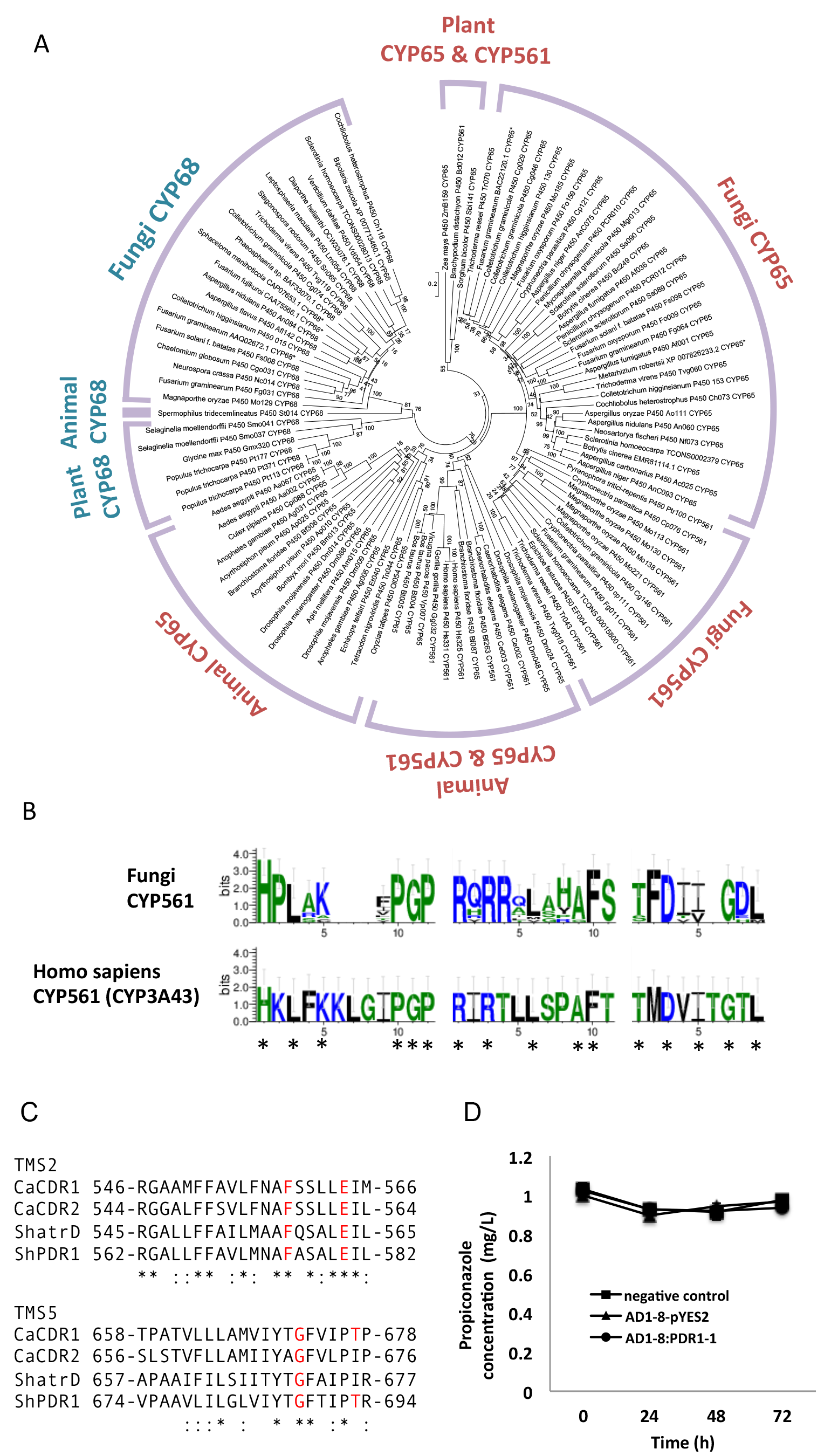

Supplement: FIG S6 [file mbo004183984sf6.tif]

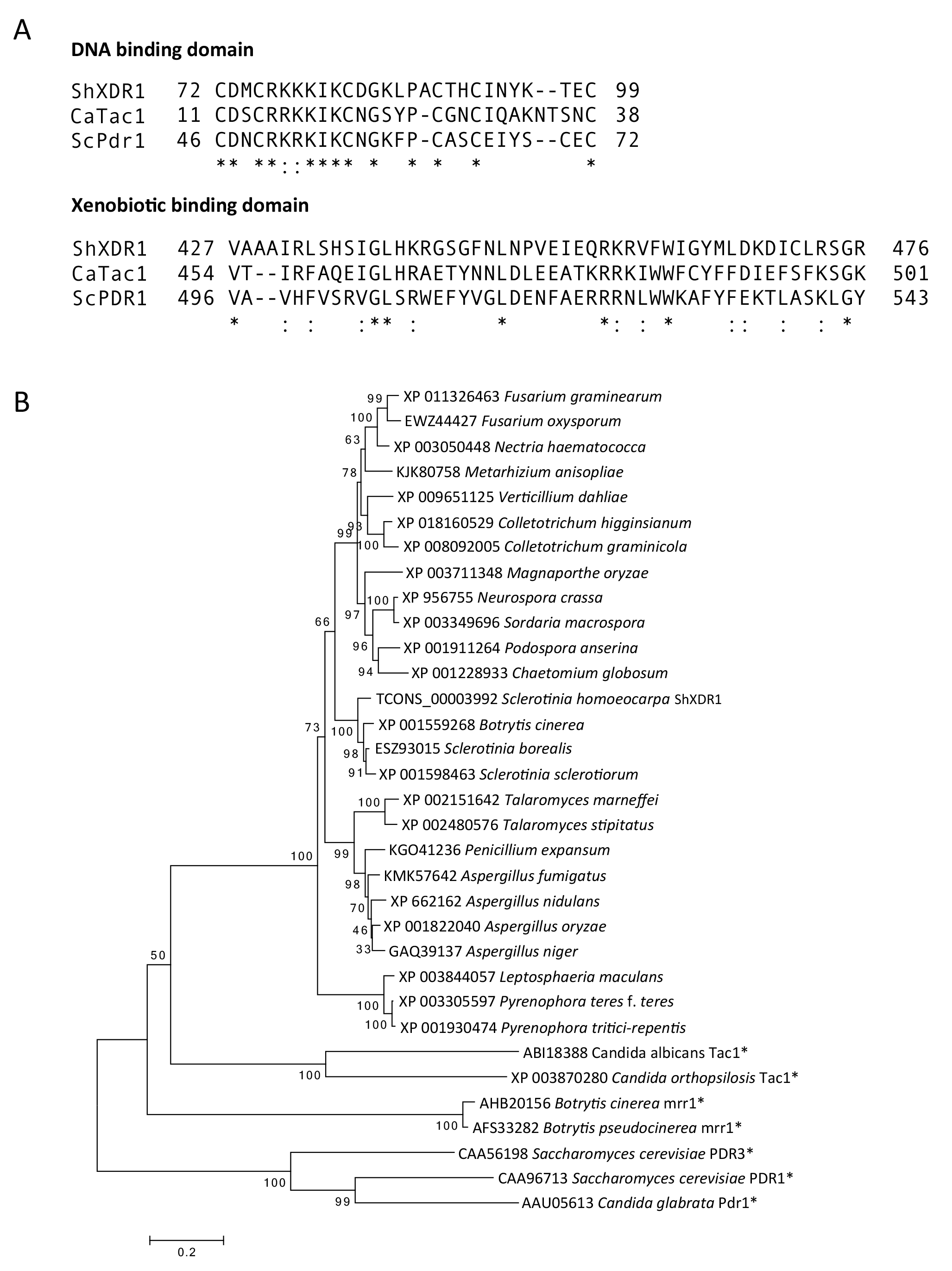

Supplement: FIG S7 [file mbo004183984sf7.tif]

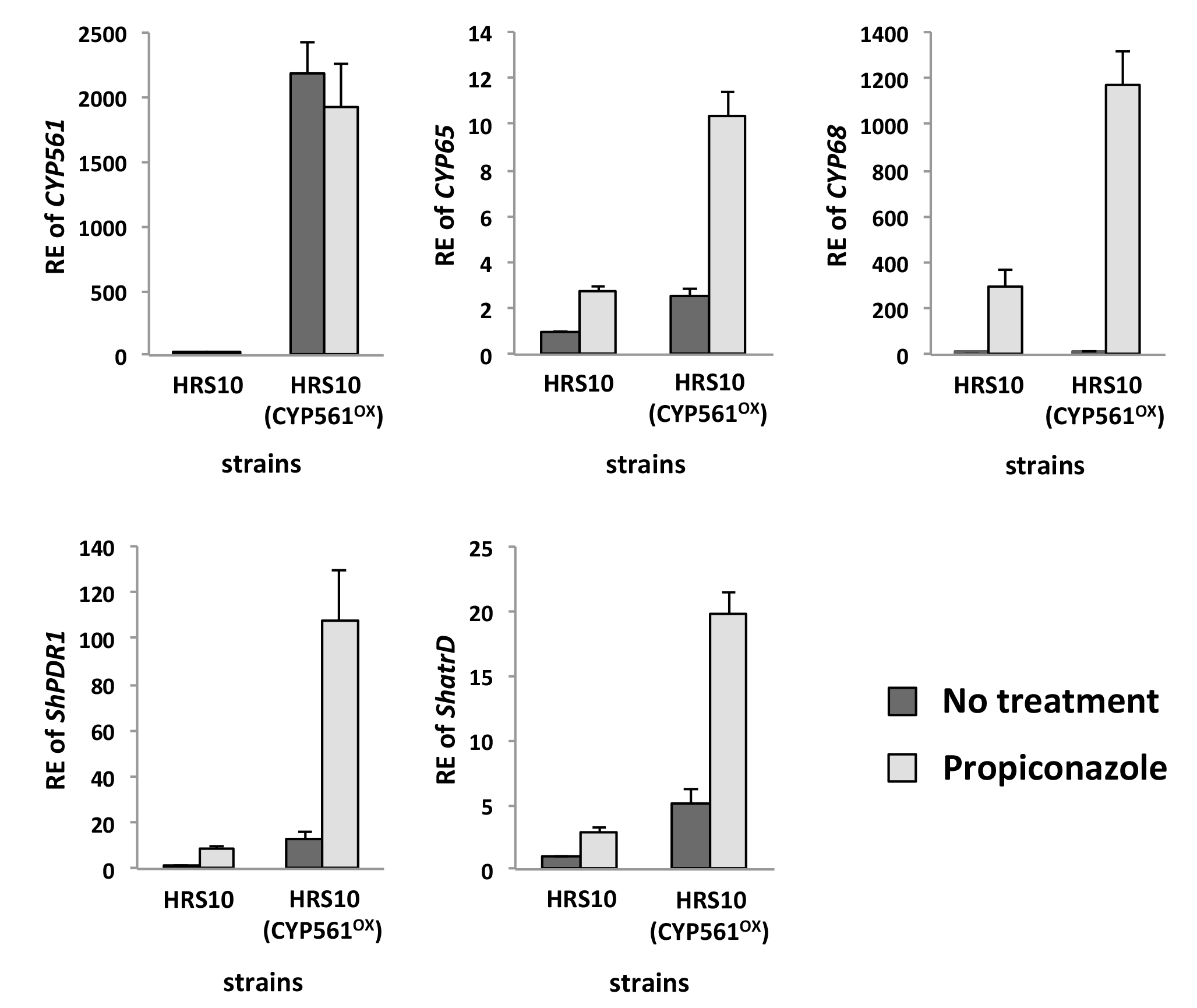

Supplement: FIG S8 [file mbo004183984sf8.tif]
