# Supplementary material for: A Xenobiotic Detoxification Pathway through Transcriptional Regulation in Filamentous Fungi
Source: mBio. 2018 Jul 17;9(4):e00457-18. doi: 10.1128/mBio.00457-18 (PMC6050962; doi:10.1128/mBio.00457-18)
Supplement: TEXT S1 [file mbo004183984s1.pdf]

Supplemental Materials and Methods

Volcano plot for RNA-seq. From the 12,008 genes identified in the transcriptomic analysis, 9,499 genes were found to have FPKM values equal to or above 0.05. These 9,499 genes were plotted using csVolcano from cummerBund, significant genes defined by $\alpha = 0.05$.

Heterologous expression of ABC transporters. The galactose-inducible expression vector pYES2 (Invitrogen) containing the full-length cDNA sequences of *ShatrD* (TCONS_00024506) was transformed into a drug-hypersensitive *Saccharomyces cerevisiae* mutant (AD12345678: AD1-8) (1) to generate two independent mutants (AD1-8:atrD-1 and -2). The ShPDR1 expressing yeast mutants (AD1-8:PDR1-1 and -2) and the control yeast mutant containing the empty pYES2 vector (AD1-8-pYES2) generated by Sang *et al*. (2) were used in this study. The yeast spot assay of the ShPDR1 and ShatrD expressing yeast mutants and empty vector mutant was conducted by the method described in Sang et al. (2). Briefly, 5 µL of cell suspension from each yeast mutant culture at 0.5 OD_{600} were spotted onto bacto-yeast nitrogen base (YNB) agar medium lacking uracil, containing 2% galactose and amended without and with fungicides and plant growth regulators (PGRs). The name of chemicals and their concentrations are as follows: propiconazole (0.005 µg mL^{-1}), iprodione (500 µg mL^{-1}), boscalid (200, 500, and 750 µg mL^{-1}), fluxapyroxad (500 µg mL^{-1}), flurprimidol (25 µg mL^{-1}), paclobutrazol (0.1 µg mL^{-1}), and fludioxonil (250, 500, and 750 µg mL^{-1}). The sensitivity of yeast mutants to respective fungicides and PGRs was qualitatively assessed after incubation at 30°C for 3 days (iprodione and fludioxonil), 4 days (flurprimidol), and 6 days (untreatment, propiconazole, fluxapyroxad, paclobutrazol, and boscalid). Two biological replicates and three technical replicates per biological replicate were performed for each mutant and treatment.

Probabilistic variant detection. To identify variants between 78 putative fungal specific Zn_2Cys_6 transcription factors from the MDR strain HRI11 and sensitive strain HRS10, Map Reads to Reference and Probabilistic Variant Detection on the CLC Genomics Workbench 6 software (Qiagen CLC Bio) were conducted. 78 transcripts from HRI11 were mapped to 78 transcripts from HRS10 and the mapped result was used for Probabilistic Variant Detection analysis. The filtering resulted in variants with following characteristics: i) no ignore non-specific matches, ii) minimum coverage =1,variant probability = 10, required variant count = 1, and iii) maximum expected variants =1 and genetic code = 1 standard. These filter criteria resulted in identifying 4 variants, TCONS_00003992 (T2558C), TCONS_00016457 (T891G, C2268G), TCONS_00003660 (G272C).

Phylogenetic Analysis. The amino acid sequences of *S. homoeocarpa* CYP561, CYP65, and CYP68, its putative orthologs in select fungal species from Basic Local Alignment Search Tool (BLAST) in Fungal Cytochrome P450 Database (FCPD: http://p450.riceblast.snu.ac.kr) and National Center for Biotechnology Institution (NCBI), CYP561, CYP65, and CYP68 in select plant and animal from FCPD, and reported CYP65 and CYP68 genes involved in biosynthesis of secondary metabolites (3-7) obtained from GenBank were used for the phylogenetic analysis. MAFFT v7.110 was used to align amino acid sequences with automated strategy selection and BLOSUM62 scoring matrix parameters (8). Neighbor-joining phylogenetic analysis was conducted using MEGA v5 with the aligned amino acid sequences with the Poisson model, gamma distributed rates among sites, complete deletion of missing data, and 1,000 bootstrap

replicates (9). For phylogenetic analysis of ShXDR1 orthologs, the amino acid sequences of ShXDR1, its putative orthologs in select fungal species identified from the BLAST in NCBI, and the reported transcription factors involved in multidrug resistance (10-13) obtained from GenBank were used for the phylogenetic analysis. The alignment and neighbor-joining phylogenetic tree were performed followed by the aforementioned methods.

Silencing of *ShXDR1* by expression of double stranded RNA. Each sense and antisense fragment (nucleotides 71-270) of *ShXDR1* amplified from cDNA of strain HRI11 was inserted into the plasmid pFGC1008 (Arabidopsis Biological Resource Center, Columbus, OH, USA). The sense and antisense fragment from plasmid pFGC1008 was inserted into plasmid pYHN3-ptrpC to generate the plasmid pYHN3-ptrpC-dsRNAXDR1 that constitutively overexpresses hairpin double stranded RNA (dsRNA) of target sequences of *ShXDR1*. The plasmid DNA (8 μg) was transformed into protoplasts from strain HRI11 using a PEG-mediated transformation system (14). *In vitro* sensitivity tests of two selected transformants (HRI11(dsRNA-XDR1)-1 and -2), HRS10, and HRI11 to propiconazole (1 μg ml^{-1}), iprodione (3 μg ml^{-1}), boscalid (1000 μg ml^{-1}), and flurprimidol (15 μg ml^{-1}) were conducted. The expression of *CYP561*, *CYP65*, *CYP68*, *ShPDR1*, *ShatrD*, and *ShXDR1* was quantified in the aforementioned four strains before and after treatment of propiconazole (1 μg ml^{-1}) for 40 min.

Knock-out of ShXDR1 by a CRISPR-Cas9 system. Two vectors p415-PtrpC-Cas9-TtrpC-CYC1t (Addgene Plasmid #68059) and p426-SNR52p-gRNA.csr-1.Y-SUP4t (Addgene Plasmid #68060) were used for a CRISPR-Cas9 system (15). The vector p426-SNR52p-gRNA.csr-1.Y-SUP4t was modified by insertion of target sequences (5’-GCGAACAGACCTGGGTATGTGG-3’) from ShXDR1 gene sequences using two-round PCR. Two fragments were amplified from the vector p426-SNR52p-gRNA.csr-1.Y-SUP4t using two primer sets (F_NheI_SNR52p and R_gRNA_XDR1; F_gRNA_XDR1 and R_KpnI_CYC1t). Two fragments that are overlapped the target sequences were used as PCR templates to amplify one fragment using F_NheI_SNR52p and R_KpnI_CYC1t. The resultant fragment and the vector p426-SNR52p-gRNA.csr-1.Y-SUP4t were digested by restriction enzymes NheI and KpnI and the purified insert and linearized vector were ligated to generate p426-SNR52p-gRNA.ShXDR1.Y-SUP4t. For the split-marker approach, two constructs (One with 1 kb of upstream region of the *ShXDR1* and 731 bp of hph and one with 1 kb of downstream region of the *ShXDR1* and 1126 bp of PtrpC-hph) were amplified using two primer sets F_upXDR1/R_YG and F_HY/R_downXDR1 from the vector Topo-ΔShXDR1 (donor vector DNA). Before *S. homoeocarpa* transformation, four different ways of DNA constructs were prepared: donor vector DNA only (5 μg), donor vector DNA (5 μg) + p415-PtrpC-Cas9-TtrpC-CYC1t (5 μg) + p426-SNR52p-gRNA.ShXDR1.Y-SUP4t (5 μg), split marker constructs only (each 2.5 μg), split marker constructs (each 2.5 μg) + p415-PtrpC-Cas9-TtrpC-CYC1t (5 μg) + p426-SNR52p-gRNA.ShXDR1.Y-SUP4t (5 μg). Hygromycin-resistant transformants were obtained 4-10 days after incubation. The hyphal-tip of transformants was transferred to PDA containing hygromycin B (100 μg ml^{-1}). *In vitro* sensitivity test of the transferred transformants to the DMI fungicide (propiconazole) was conducted. The transformants and strain HRI11 were grown on PDA for 3 days and an agar plug (5 mm) of each strain from the edge of colonies was placed on PDA and propiconazole (1 μg ml^{-1}) amended PDA. After 2 days of incubation at 25°C, the number of transformants grown or not grown on propiconazole amended PDA was counted. The expression of *ShatrD* was quantified in strains HRI11, Trans1, and Trans 2 before and after treatment of propiconazole (1 μg ml^{-1}) for 40 min.

REFERENCES

1. Decottignies A, Grant A, Nichols J, de Wet H, McIntosh D, Goffeau A. 1998. ATPase and multidrug transport activities of the overexpressed yeast ABC protein Yor1p. J Biol Chem 273:12612-12622.
2. Sang H, Hulvey J, Popko JT, Lopes J, Swaminathan A, Chang T, Jung G. 2015. A pleiotropic drug resistance transporter is involved in reduced sensitivity to multiple fungicide classes in *Sclerotinia homoeocarpa*. Mol Plant Pathol 16:251-261.
3. Hu X, Xiao G, Zheng P, Shang Y, Su Y, Zhang X, Liu X, Zhan S, Leger RJ, Wang C. 2014. Trajectory and genomic determinants of fungal-pathogen speciation and host adaptation. Proc Natl Acad Sci U S A 111:16796-16801.
4. Bömke C, Rojas MC, Gong F, Hedden P. 2008. Isolation and characterization of the gibberellin biosynthetic gene cluster in *Sphaceloma manihoticola*. Appl Environ Microbiol 74:5325-39.
5. McCormick SP, Harris LJ, Alexander NJ. 2004. Tri1 in *Fusarium graminearum* encodes a P450 oxygenase. Appl Environ Microbiol 70:2044–2051.
6. Kimura M, Tokai T, O'Donnell K, Ward TJ. 2003. The trichothecene biosynthesis gene cluster of *Fusarium graminearum* F15 contains a limited number of essential pathway genes and expressed non-essential genes. FEBS Lett 539:105-10.
7. Tudzynski B, Hölter K. 1998. Gibberellin biosynthetic pathway in *Gibberella fujikuroi*: evidence for a gene cluster. Fungal Genet Biol 25:157-170.
8. Katoh K, Toh H. 2008. Recent developments in the MAFFT multiple sequence alignment program. Brief Bioinform 9:286-298.
9. Tamura K, Peterson D, Peterson N, Stecher G, Nei M, Kumar S. 2011. MEGA5: molecular evolutionary genetics analysis using maximum likelihood, evolutionary distance, and maximum parsimony methods. Mol Biol Evol 28:2731-2739.
10. Kretschmer M, Leroch M, Mosbach A, Walker A-S, Fillinger S, Mernke D, Schoonbeek H-J, Pradier J-M, Leroux P, De Waard M, Hahn M. 2009. Fungicide-driven evolution and molecular basis of multidrug resistance in field populations of the grey mould fungus *Botrytis cinerea*. PLoS Pathog 5:e1000696.
11. Thakur J, Arthanari H, Yang F, Pan S-J, Fan X, Breger J, Frueh D, Gulshan K, Li D, Mylonakis E, Struhl K, Moye-Rowley W, Cormack B, Wagner G, Näär A. 2008. A nuclear receptor-like pathway regulating multidrug resistance in fungi. Nature 452:604-609.
12. Plesken C, Weber RWS, Rupp S, Leroch M, Hahn M. 2015. *Botrytis pseudocinerea* is a significant pathogen of several crop plants but susceptible to displacement by fungicide-resistant *B. cinerea* strains. Appl Environ microbiol 81:7048-7056.
13. Coste A, Turner V, Ischer F, Morschhäuser J, Forche A, Selmecki A, Berman J, Bille J, Sanglard D. 2006. A mutation in Tac1p, a transcription factor regulating *CDR1* and *CDR2*, is coupled with loss of heterozygosity at chromosome 5 to mediate antifungal resistance in *Candida albicans*. Genetics 172:2139-2156.
14. Sang H, Popko J, Chang T, Jung G. 2017. Molecular mechanisms involved in qualitative and quantitative resistance to the dicarboximide fungicide iprodione in *Sclerotinia homoeocarpa* field isolates. Phytopathology 107:198-207.
15. Matsu-ura T, Baek M, Kwon J, Hong C. 2015. Efficient gene editing in *Neurospora crassa* with CRISPR technology. Fungal Biology and Biotechnology 2:1.

Sequence information

>upstream_ShXDR1_HRI11
TTGTCATCTCTTACGGGAATTTTAATGATCTGTTGGATAAATAGACGCCACCTTGTAGT
GTTGGGCGAAAGGCCAGGGCCTGATATCAGATGTACCGATAAAAGATAATGTCTTTCC
ATGTATGAAACCTCGGATGTAATTCTTGAAGTCTTAGTGCATTGAGCGTTGTGATGTTC
TCCCAATTCGGCCATTTCAAAAGGATGATGGAGCGTAGAGCTTGGTCTTACACAAAA
CACGATGGTGACTATGTATGTGAGAACCTGTGAGGATTCATGGCTTTGGGTCCATGCG
GGGAGCTTCAAAGCCCGAATTGGTAATTGTGATAATCTCCTTATCGCCGAAGAAAGTC
TTTCCGCTTGTTTCAGTAGTGTCACAGATCGAACCTGGAAGGTACTCATTGGCCAAAG
AAGATTTGCATCGACCCCACTGAAGTCGATGGCGTCTATCTAGAATGTCACATCAATAT
GCAAGTTGACGATTACCAAGGTTTGGTCACAAGTAGCAAGTAGAACGAAGAATGAAT
GAGAATATACGGATGATCGTTCCGAATATCTCGAAGTATGCAAATCATCAGTCTCGTGT
GCCATGCGAATAAGGAGATGTTCGTCCTCATGTGTTGAATTTTGTAACTCTTTGCAGCT
CAGTATCGATTCCCAGTTCAGCCCGCTTTAACTTTATTTCGACATCTCTAGTAGATGTTT
GGTGTGAGAGATGACGCCGTACTTGTCCTGCTGAGGTATGGAATGAGACGAGGGCAA
CAGACTGTGTCAGATATTGTTTCCTTTTTATACCACTGGTGTAGTAGTATATATTTCCGG
CACATTACATTGTAGCCTGCGCCCCTATTTCATCTCCTCAGGTCTTGCAGGAGTCTAGA
TGTATGCTTATGAAGAATCGTTGCTCCACGGATCAGTAGTCGTATTGGATATCTTTTGC
GCCGACTTAGCTGCTCCCCCCTACTCACCTGCCATGCTACGCATGTAACATTCGATGTT
GTGAAAGCAGAATTTCTCTCAGCGATTCTTCTATCCGTCTAAAATATTAATGCTTGTCT
AATTCTTTTACATTCTTTTACATTCTGAGTGAATTTCTGACTAGAACATTAATAATAGTC
CATTTTGCGTCAAGTAACCGCTTTTTAGTTGCAGCACCCTAATTCCCACCTCCGAAAC
GGAGGACCAAACCAACGACCCCTCCAATTCCACAGTACAGTCAGTACTCGCTGCCAG
GTACCTTGAGCTTCCGAGCCTGGTTGCGACTTTTACTTTCAGCACGACGACACACCTG
AGAGCAGGTCAACCTTTGTAAAGATCTGTACTCGTCATCAGCCCCGCCCGCAGCATTC
ACAATCAATACCTTGTGCACAAAAACACCAGGGGAGCCAAGGCAGTTGGGTGGAAA
CATTGCGGAAATTCAGCCATACTCCGTGTCCTAGCGATTGTGACCTCACGAAGAGTTG
TACGAATACGAAACTGCATCATTCCTGGCAGTGCACAGGTACTTTGACCGGATTTCGA
AGGACTTAACGCAGTCTCTCACAACGTCAACAATACACTTCAAAATACTATGCGATAA
TTAGTG
>ShXDR1_HRI11
ATGATGGACTCCATGCATCGTGAAAGGAATTCTTTCATTCCCCAGATTTCAGCGCCGC
CTTTGATGAACCCCCCACCACAGATCTTCGGGGCTTACACTGCAGATGGCATGCCGAT
GTCCTCGACGTTACCTGACATGAGTGGGCCGCTTTTTGGAGATGGCACCCTTCTCGAC
GAGAGTAATGAAGCGAAGCGGAGAAGAATAGCTAGAGTACGACAAATGCCTGATGG
AAGGAGAGTGGTTCATGACTGACGATGGCAGGCTTGTGATATGTGTAGGAAGAAAAA
GATTAAATGCGATGGAAAACTACCCGCTTGTACGCACTGTATCAACTACAAAACGGAA
TGTGTTTTTACACAAGTTGAGAAGAAGAGGAACCCGCCGAAAGGGTAAGCAAATTG
AGAACAATACACAGTATATCGCTAACTCCTGGACAGAGCAAAATATATCGAAGGTCTA
GAAAATCGTCTGGGACGCATGGAAAGTTTGCTGCGGCTGTCTGGTTTGTCTACCAATT
CCCATGCGCATAAGAGCCGACAACATCATAGTTGCTCACACATTTCAAAGGACTCCTC
AACGAAGACGATAATGGGCGAACAGACCTGGGTATGTTGGAGAAGAAATTACAGGAT
AGGAATCAATCACGGAGAACATCTGCTGTACCAGGATCAAATCCCACATCTCCGGAA
CAAAGTACCCCAGGCCATAACGACGGCAATGCCTCGACACCCCGCAGCGCCATGGCA

TCACCAGAACCGCAAAGTAAAGATAAGGAGAAGCGAAATAGCAAAGATAGCAAAGA
TGGACGCAGTGAGAATAACGAAGAGCCGGTAGAAGCACTTTCTGATATGATGTGTTC
CCTGGTTACAAATAACTGTGGAGAAACTAGATACATAGGTATGCAGTGGACTGCATAG
CAGCGGACTCTACTGACTGTGATAGGATCATCTTCCGGCTTCTCTATCTTCTCCCCGAA
AGGTATCCAATGGGTCAATGAAAAAACCGGCGATTCGTCCTTCCAAGAAATGATATCG
TCCGCTTCAGTCGACGACAATAAGTCGATGTACTGGAAGCCAGAAGTGTTCGCTGAT
TTGTTCCACCGACCAGTCTTCAAACAGCTACCCCCCAAAAACGAATGTATATCACTGC
TGAAAGACTTTTTCGAGAATTTCAATTGCATGTTTCCGCTTTTTCATCAACCAACGTTT
ATGCATCTTGTCGAACGGCAATATTCCCCCGATCCATACCAGGGTTCTGGATGGTGGG
CTAGTCTCAATTGTGCGCTTGCTATTGCTCATCGCCTTCGTGTTATGAGCCACCTAGTA
CCAAAAGATGAAGATACAAAGGCCTGGGGTTACCTGAAGAATGCGATGGGTGTATTT
ACGGAATTAACTATGCGGAACACGGACCTTCTCAGCGTGCAGGCACTTTTGGGCATG
GCATTGTTTATGCAGGGCACGCCTAATCCTCAGCCATCCTTCTTTCTTGTAGCAGCTGC
CATTCGGTTATCGCATAGTATCGGACTCCACAAACGAGGTTCTGGCTTCAACCTGAAC
CCGGTAGAGATCGAGCAACGAAAACGAGTTTTCTGGATTGGTTATATGTTAGACAAG
GATATCTGTCTGCGATCTGGACGACCGCCTGCACAAGATGACGATGATATGAATGTTG
AGCTACCTAGCGCTGATCCAGATGACAACATTGGCAATATTCCTTTGGCAGATGGAAA
GGGCAAGGTCAACCTTTTCCGATTGATGTGCGAATTTGCGACCATTGAAAGCAAAGT
CTACAAACAACTTTACTCTACCAAAGCCTCGAAGCAGTCCGATGGAGAACTCCTAAA
CACTATCGGCGAGCTAGATCACGAGCTTGAAGAATGGAAAGATAGCATTCCCGTTGAT
TTCCGCCCAGAACATGAGATTAAAGCTTCACACACACCCTTGATCCTCCATGTTGTCG
TCCTTCATTTTGCTTACTACAACTGTCTTACTACTATTCATCGAATGTCTGTACATCATG
GGTACTGGACAAGTCGACTTTCCAATTATGCTATTCAGGGTCTCAACGCTAGCCCCCT
AAATCCTCGGGTATTTTCTTCTGCAGCTTTGTGTGTTTCTGCTGCTCGCGCCTCCATCC
ATCTCATCAAATATGTTCCTCAGGGAGACTTCGCTTGTGTCTGGTAAGTCGCATGACA
ACCGGCTATGTTGGGGACTAAACTAACAACCGTAGGCTTATTCTCTACTTTCCTGTTTC
TGCATTAGTGACTCTTTTCGGAAATATTCTACAAAACCCTCAAGATCCAAGGGCTCGC
TCTGATGTACGCCTGATGAACCTTGTTGTAAACTTTCTTTCTATGCTAGGCGCCGAAG
AAGAAAACGGAGGCGTCAAACGCATGCTAGGAGTCTGCTCAGAGTTCGAGAGGATT
GCAAAGGTCGTTCTCGACAAAGCGGAAAAGGAGACATCTTCGAGGAGGAAACGCAA
GAGCCATGATGGTCATACCAAATCCTCACATATCTCGAGAGCCAGCCAACCGCAGAC
ACCAATAATGAACCACTCTCAGACACCAATGTCTGGAGTCATCTCGCCTAACATGTCC
AGCCATCTTAATCCTCATAATGGTTTCAGTCCCATTCAAGGCTCGCCGAATTCAAATTG
GCAGCCGGATTTACACAGTACTGGAGGTGATTATATGGGGTCAGCTGGCACAACATTT
GAGGACATGCACGGGTTTACAAATGGCAACGATGCCAATTCACCACTTAATCTTAATG
CTTTCCAGCAACCATTTGTTCCACAAGATTTATGGCAAATGCCCACGACCATAGAATG
GGACTGGGCTGAAATGACCGGAGGCGCATACCAAAGTTTCGAGAATGGAATTGTGGG
TGACCCGGAGCTGCATATGAACAACAATTTACACTTGCAACATCAAGGTGGAATGTAA
>downstream_ShXDR1_HRI11
GAGTTCGGTTTTAGCATTGTATATTTTGGGCTGCGAGGCAACGGTGTTCtAGGGGCATT
TGACACTAAGGACGATGGATCATGAAGAACGGATTAACGGCTTAGGAAGCAATTGCC
ATTGCATGGAATGAGGCGCATTTACTAGAAACTCGTTTGGCTCGAGATGTTtGCTTTTT
CCCTCTCGTAAATCGGTGGGAGGGTGGGAGGATGGCATGGCTGTACACTAACTATGA
CTCTCCTCAGATATTTGTACTGCGGCCCTGAGGGGCTTGCCCAAGCACTGTAGTAATG
ATATATTGGTATTATAAACCCGCAGATTGCTCTTCAATTATGATAATATTTTAAGTCTAAA
AACTGTGTCTACACATATCAATCACTTGAATCACATCATTAATATTATAAGGATAAAAA

GCTTGGTTTGTAATTGAGGGCATGTCAAAAGCTCGTTGGATGTATTTTGCTTCTAGACC
TGACAAAGTTGCGCTAGTGGTCGCTTTCACTAATCAAGTGGCCCTGCACATAATCAAG
ACATCATTGCGGAAATCGAACCGACATTGCCTTCCACCACTAACTCCGAAGTCAACG
AGGACCTCTTCCAATCTATCCCGCGAGTCTATGAAATAGTGGTCAAAGTCACTGAAAG
ATACGAGACAGTGACTTGAATCCTCTTCTTtCCTTTCAAATACAACCAGCAGCTCTAGC
CAAGCATGACATCCATGTTACGGCAATCGAGCAATCTTGCTAGAATTTCTCATGCCAC
CATTCAATCCGCTGCACGGAGTTCCCTTCGCACATCATCTCATGCGCCTCTACTGAGTT
CGCGGTTACTCGCACAAAAGGTGCGCGCCTACTCTTCGAATTTAATCAACCAAAGA
AAGATGGACGTGAGGTAGAATCAAGGAAGAAGACGCCTACGCCGTCTTCCAAGTCG
GCTCCTGGGACGGAAAATCTTTACAATGGAAAAACACCTTTGTCGAACCGGGACGAG
AATGCGAAGAGTTCAGAGGAAAATGAGACACCATTAGATGAAGGCATGGTTAGGTTG
ACGGAGGAGGAGCTGAAGGAATTAGAA
>ShatrD_HRI11
ATGGCGACCTCGACACCCTTTGAGGTTTTGCCTCAAAGTGAGGCAGTGGCCCATAAA
ATTGAGGAGGTTGTTGAGTCGAAAGTAGAGACATCTTCCAATCATGAGCGGAAGGTC
AGTGATACAGGCTCCTCGACGTTGGGTTCCATTGCAGAAGAAGATACTGCTGTTCGTG
AACTGGCCAGACGCATCACTCAACAGTCAAGGGGATCCGACGATGGCCAGCATCATA
TCAATCCTTTCTTTGGATCGGATGATCCCACTCTCGACCCATCATCTGAAAAGTTTAAC
TCTAGAGCTTGGGTTAAGAATCTTGTTCAAATCCAGTCTAGGGACCCCGAAAGATATC
CTAATCGAACAGCCGGAGTCGCCTACAAGAACTTGAACGCTCACGGATTCGGAGTAG
CAACGGATTACCAGAAGACTTTCGGAAACTACCCATTGGAAATCGCTGGCATGGCTA
AAAGGATTCTTGGTGTCAGCAAGCAAACCAAGATTCAGATCTTGAGGGACTTTGATG
GGCTAATCAGAAGTGGCGAGATGCTTGTTGTGCTCGGAAGACCAGGTTCAGGCTGCT
CAACTTTACTCAAGACTATCTCTGGAGAGACTTCGGGTTTCCACGTTGACAAGGATAC
CTACATTAACTACCAAGGTATTCCAATGAAGACAATGCACAAGGATTTCAGGGGAGA
GTGTATCTACCAAGCAGAAGTCGATGTTCACTTCCCTCAATTGACGGTATCGCAAACT
TTGGGCTTCGCAGCACAAGCTAGAGCCCCTCGAAACCGTATGCCCGGAGTATCGAGG
AAAGTTTACGCTGAGCATCTGCGCGACGTCATTATGGCAACCTTCGGACTTAGCCACA
CCTTCAACACTAAAGTTGGCAACGATTTCATTCGTGGTGTCAGTGGTGGTGAAAGAA
AGCGTGTCAGTATCGCAGAAGCAGCCTTGGGTGGCAGTCCTCTACAATGTTGGGACA
ACAGTACTAGGGGGTTTGGATTCAGCGACTGCTTTGGAATTCGTTAAGACTCTTCGAAC
ATCTACAGAGATGACTGGTTCTACTGCTATCGTGGCTATTTACCAAGCTTCTCAATCTA
TCTACGACATATTTGACAAAGTAGCGGTTCTATATGAAGGACGTCAAATCTATTTCGGA
AATATTCATGCGGCTAAGGTATGTGGTTTAGATTTTCATTACACTTCGAGATTCCTTTTG
CCTTTCTTAATATGTGTGATATTTGCGTTTCACCCCAGTCGAACAAATGGATGTAGTCA
TGTCCAATGATATGATTGTGTGATAGAAATGGACTTGTCTATCAAACAAAAAGTGCTAT
AGTACAGTACTGTACAGCATCATGGAGAACGATTGGAACGAGAGATCAGTGATCTGAT
AAAAGAAATCTTTCCGTGATCTTGGTTGGGAATTCACTAACATAGATGCGTTTTTTTAG
ACCTTTTTCATCAACCTAGGATTTGACTGCCCACCGCGACAAACTACAGCCGATTTCC
TCACTTCTCTTACTAGTCCCGCGGAACGAATTGTGCGACCTGGTTTCGAAGGCAGAA
CTCCCTATACCCCTGATGAATTCGCCGCAGTTTGGCAAAAGAGTGAAGATAGAGCGC
AACTACTTCGGGAGATCGATGAGTTTGATGCTGATTATCCCCTTGGTGGTCCATCGCTC
GGGGCTTTCAAAACATCCAGGAAAGCTGCGCAAGCCAGAGGCCAGCGTCTTAAGTC
GCCTTACACAATTTCGGTACCTATGCAGATTAAGTTATGTTTGGAGAGAGGTTTCCAA
CGTCTACGTGGTGATATGACGATCTTTTTGAGCGGTGTTATTGGTCAATGTGTCATGGC
ATTGATTCTTGGTAGTGTGTTCTACAACCTTTCGGACGATACCAACAGTTTCTATAGTC

GAGGAGCTTTATTGTTCTTTGCTATTTTGATGGCTGCTTTCCAGAGTGCATTGGAGATT
TTGACCCTCTATGCGCAACGACCGATTGTGGAGAAACATACGAAATACGCCTTTTATC
GTGAGTACTGCCAATTTCTAGTTCCATTCTTTCTGGTGTCAAATCTTCCCTTCACATTT
CTTGGCTTCTAAGACATCGATTTTCGCTAACCATCTTTTCCAGATCCATTTGCTGAAGC
TTGCGCATCCATGTTGTGTGACTTGCCTAACAAGATTTGTACTGCTATTGTGTTTGACC
TTGTGCTCTACTTTATGACCAATCTACGAAGGACACCGGCGAATTTTTTCGTGTTCTAC
TTGTTCACTTTGGTTTGCACACTCACGATGTCCATGTTTTTCCGCTCGATTGCTGCACT
GTCACGGAGTTTGTCAGAGGTAAGCCGCCATCCCTTCTGTGCTTGCTACCCAAGCTAA
TCCAAGTGCAGGCCATGGCACCTGCCGCAATCTTCATTCTTTCGATTATTACATATACC
GGTTTTGCTATTCCAATCAGAGATATGCATCCCTGGTTCCGATGGATCAATTATGTCGAT
CCAGTCGCTTATGGATTCGAAGCACTCATGGTTAACGAATTTCACAATCGAAAGATCC
CTTGTTCAGTGTTTGTGCCATCCGGTCCTGGTTATGGAAGTGTTTCTCCGGAGCAGAA
GATTTGCTCCGCCACTGGTGCTGCTGCCGGAGCTGACTTCGTTGATGGTGATACATAT
CTTGAGGTCAACTTTGGCTACAAGTACAGTCATCTTTGGAGGTAAGTTGATTCTGGAC
ATCCGAAATTTAAATATGCGACTTATGGTTACACAGAAACTTGGGTATCATGATAGCCT
TCACTATCTTTGGAATGGCTGTTTACCTCACCGCAAGCGAATTCATCTCTGCAAAGAA
GTCCAAGGGAGAAGTGCTACTTTTCAGGCGTGGACGTGTACCCTATGTTTCAAAGTCA
TCTGACGAGGAATCAAAGGGTGAGGATCGTATGACCACAGAAACTGTAACTCGACAA
AAAACTGTTCCAGATGCGCCCCCAAGCATTCAAAAGCAAACTGCGATTTTCCATTGG
GACGAAGTCAACTATGATATCAAGATCAAGGGCGAACCCAGAAGGCTGTTGGACGGT
GTTGATGGCTGGGTTAAACCTGGTACTTTGACTGCTCTTATGGTAAGTTCTCTATGATT
CTTCCTCGGTCGAATGGCACGAATATCATTCCTTATATCTCATAAATGATGTACAACGCT
AACTCATGATTTAGGGTGTCTCTGGTGCCGGAAAAACTACTTTACTTGATGTGCTCGC
TTCTCGTGTGACTATGGGTATTGTTACTGGCCAAATGCTTGTGGATGGTAAGGAGAGA
GATATTGGTTTCCAAAGAAAGACTGGTTACGTTCAACAGCAAGATCTTCACTTGGCTA
CTTCCACTGTTAGGGAAGCTTTGACTTTTAGCGCTATTCTGCGACAACCGGCCACTAC
CCCTCACGCTGAAAAGGTTGCTTATGTCGACGAAGTTATCAAGGTCTTGGAGATGGA
GGCTTATGCCGATGCCATCGTTGGTGTTCCTGGTGAAGGTAGGTTATACCTTGGGCGA
CTCCAATTACTTAAACGTTCTAACATGATTTCAAAGGTCTTAACGTCGAACAACGTAA
ACGTCTTACAATTGGTGTTGAGCTTGCCGCGAAACCTGCCTTACTTTTGTTCCTCGAT
GAACCAACATCTGGTCTTGATTCTCAGACTGCCTGGTCTATTTGTGCTTTGCTCAGAA
AACTCGCAGATAATGGACAGGCTATTCTTTGCACTATTCATCAACCTTCCGCTATTCTC
TTCCAGGAATTTGATCGTCTCTTGTTCTTAGCCAAGGGTGGTCGAACGGTATACTTTG
GTGAAATCGGCAAACACTCGAAAACCTTGACAAACTACTTCGAACGAAATGGTGCCC
ATCCTTGTGGTGATGTTGCAAATCCTGCTGAATGGATGCTAGAAGGTAAGTCTTTCTAT
CTTGTTGAATTTTAATGCAAACCTAACAATCAATTAGTTATTGGTGCTGCGCCTGGATC
TGAAACTACTATCGACTGGCCACAAACTTGGAAGAACTCACCTGAACGTCAACAAGT
CAAAGCTACCTTGGCAGAAATGAAGCAGACTCTTTCTGCCAAACCAATCGAGCATGA
CCCCAATGCACTCAACTCTTTCGCTGTCGGCTTCATGACGCAAATGTGGGTCGTGCTC
CTACGTGTATTCCAACAATACTGGCGTACTCCATCCTACCTCTACTCAAAAACTCTTCT
TTGCACGTGCGTTGGTCTTTTCATCGGTTTCTCATTCTGGGATACAAAGACATCTCTTC
AAGGCATGCAAAATCAACTTTTCGCTATTTTCATGCTTCTTACTGTAAGTCTCGATATC
AATCTTCCAACTTTATAAGCAAAGACTAACCTGGAACTAGATCTTCGGTAACCTGGTC
CAACAAATCATGCCTCATTTCATTACTCAACGTTCTCTGTATGAAGTTCGCGAACGCC
CATCAAAGACCTATTCATGGAAAGTTTTCATCCTCTCCAACATCTTTGTTGAGTTGCCC
TGGAATACCCTCATGGCTGTTATCATTTTCGTCACATGGTACTATCCGATCGGTCTTCA

AAGAAACGCTGAAGCAGCTGGTCAAATTACTGAGCGTAGTGGTTTGATGTTCTTGTA
CGTTTGGGCATTCTTGATGTTCACTTCTACCTTCACTGATATGGTTGTCGCTGGCATGG
AAACTGCCGAGAATGCTGGAAACGTTGCCAACTTGTTGTTTACTCTTACATTGATTTT
CTGCGGGTATGTTCCTCTCTCCTTTTTGTCGAAGTAATTAGTTTACTAATCCCACCACA
GTGTGCTCGCAAGCCCAACCTCCCTTCCCGGTTTCTGGATCTTCATGTACCGCGTATCT
CCCTTCACTTACCTCGTTAGCGGTATCATGGCCACCGGCCTCGCCAACACCAAAGTCA
TCTGCTCATCTATCGAATACCTCCACTTCAACCCCCCCTCTTCGCAAACATGTGCCGAA
TACTTGGACCCGTATATCTCATTCGCTGGCGGCTACTTGAATAATCCCAACGCGACTAC
CGATTGCGAGTTCTGCTCTCTTAGCGATACGAATGTTTCTTGGCGAGCGTGAATAGTT
ATTATAGTCAGCGCTGGAGGAACTGGGGGTTGCTGTGGGTGTTTATTCTCTTCAATATT
GCGGGGGCCATTGGGCTGTATTGGTTGGCTAGGGTGCCGAAGGGGGCGAGTAAGGG
GGATAAGGAAGAGGGTGCGAAGGTGGAAGGGGAGAAGAAGAAGTGGTTTAGGAAG
AAATAG
>ShPDR1_HRI11
ATGTCTTTATTAGGGAATCTCAACCCTAATCTCAACCCAGGGCAATCCACGGTTAGCC
ATAACATTCCATCCCGCCAATTCCACGATGGGGAGGAAGACATCGAGCTTTCCAATGT
CAACAAGAGCTACGCCTCCAGCGATACAGCCATCGTGGGCAAGAACGAAGAAGAGG
ACGGTGCATCCCATATTCCGACTGAAGAACAGCTCAATGCTGATATCGGTCGTTTGGC
TCGACGACTGACCCAGCACTCAACACGATACTCAACCAATACGAATCTTCCGAACCC
CTTTCTACTCGACAAAGACGATTCGACTTTGAACCCTCACAGTCCAAATTTCAAAGCA
AGTGACTGGATGAAGATGTTGCTTGAAATTCGATCGAAGGATCCTGAGAGATATCCAA
AGGGAACAGCGGGTATTGCGTTCAAGAACTTATCTGTGCACGGCTTTGGCAGCCCCA
CCGACTATCAGAAGGATGTCATTAATACTGTTTTGGAGCTTGGTACTTTGGCTCGTCG
AGCAATGGGGATCAAGCCGCAGAAGATTCAGATCCTTCGCGATTTCGACGGTTTGGT
ACGGAGTGGAGAAATGCTGATTGTCCTTGGTCGACCTGGGAGTGGATGCACGACATT
GTTGAAGACAATTGCTGGAGAGACAGGTGGTATTGAAATGGATGAGAAGACCGTTTT
GAATTATCAAGGTATATATATCCCTTTTATCCTTTTGTTTGGTTTCAATCGATGGAAATC
GTGTTTGTGACTTACATTCAACGAAATAGGCATATCAGCAAAACAAATGCACAACCAA
TTCAAGGGCGAAGCCATCTATTCCGCAGAAACCGACGTTCATTTCCCACAGCTTTCTG
TTGGTGATACCCTCAGCTTTGCCGCACTTGCACGTGCCCCTCGAAACCGATTGCCCGG
GGTCAGCCGAGAACAATACGCTGAACACATGAGAGATGTGATCATGGCTATGCTGGG
TCTCTCTCATACTGTCAATACCCGCGTAGGCAACGACTATGTTCGTGGGGTATCTGGTG
GAGAGCGCAAACGTGTTAGTATTGCTGAGGCTACGCTCAGTCAAAGTCCACTGCAGT
GTTGGGATAACAGTACCAGAGGTCTCGACTCCGCCAATGCCCTCGAGTTCTGCAAAA
CACTCGCTCTAACAGCCAAGTACTCTGGTAGCACAGCCTGTGTTGCTATTTACCAAGC
CTCGCAGAGTGCTTATGATGTCTTTCACAAAGTCACTGTGCTCTATGAAGGACGACAG
ATCTTCTTTGGCCGAACCGACAAGGCCAAGGAATTTTTTGTGAACATGGGATTCGAGT
GCCCAGAACGCCAAACCACAGCGGATTTCCTAACATCTCTCACCAGTTCCTCAGAAC
GTGTTGTCCGGAAAGGCTACGAAGGCAAGGTTCCTCGCACACCTGACGAGTTTGCGG
CTGCATGGAAGAAGAGTGACGCGTACGCCGAGCTCATGAAGGAAATCGACGAGTAC
AACAACCAATACCCTATTGGCGGCGAGGCATACGAGCAATTCGTTGAGGCTCGCCGC
GCAGCGCAAGCTAAGCACCAGCGGTTGAAGTCTCCTTTTACAATTTCCATGGCGCAG
CAGGTCAAACTGTGTGTTACTCGTGGTTTCCAGCGCCTCAAGGGAGATGTCAGCTTG
ACGATGAGTGCTCTTATCGGTAACTTTATCATGTCCTTGATTATTGGCAGCGTATTCTAC
AATCTTCCTGAATCGACTGCAAGCTTTTACTCTCGCGGTGCACTATTATTCTTCGCCGT
CCTTATGAACGCTTTCGCTAGTGCCTTGGAGATTCTCACCCTCTATGCCCAGCGGCCA

ATTGTGGAGAAGCAGGCCCGATATGCCTTCTGCCACCCCTCTGCAGAGGCGCTTGCG
TCTATGATTTGCGATATGCCATATAAGATCGTCAACTCTTTCACGTTCAATATCCCATTG
TATTACATGGCCAATCTGCGCCACGGCGGTGATGTCTTTATGACATTCTGGCTCTTCTC
ATTTGTTGCAACTCTCACCATGTCTATGATTTTCCGAACTATCGCTTCTGCATCTCGCA
CTCTATCACAGGCTCTTGTGCCCGCTGCGGTATTGATATTGGGCTTGGTCATCTACACT
GGCTTCACAATTCCTACTCGAAACATGCTGGGTTGGTCAAGGTGGATGAACTATATTG
ATCCGCTCGCGTATGTCTTTGAAAGTTTGATGGTAAATGAATTCAATGGTCGGACATTT
CCTTGCTCAGCCATTGTCCCATCTGGAGGCAGCTATGACGGTATATCAATGGACTATCG
CATCTGCTCTACTGTAGGTGCTGAAGCAGGATCAAATGTTGTCGAAGGTGCCAGCTAT
CTTGCCCAGAGCTTCCAATACTACAAAGGACATGAATGGAGAAACTTTGGGATCATCA
TTGCATTCATGCTCTTCTTCATGGCTACCTATCTTCTTGCTACCGAATACATCTCCGAGC
AGAAGTCCAAAGGCGAAGTTCTCTTGTTCCGCCGCGGCCACCAGCCTGCTCATGTCG
GATCGGATGTTGAGGGTGGTGTGACCGAGACTACGAAAACAGCTGATCAACAAGGC
GGTGCTGCGATCCAGAGACAAACAGCTATCTTCCACTGGCAGGATGTGTGCTACGAT
GTTAAGATAAAATCGGAAACCAGACGTATTCTTGACCACGTTGATGGATGGGTGAAG
CCTGGTACTTGCACAGCATTGATGGGTGTCTCAGGAGCCGGTAAGACGACCTTGTTA
GACGTCCTCGCCACCAGAGTAACCGTGGGTGTGGTCACAGGCGAGATGCTTGTCGAT
GGCAAACCACGCGACCAGTCATTTCAACGGAAAACCGGCTACGTACAACAACAAGA
CCTACACTTATCCACCTCGACGGTCCGAGAAGCTCTAGAATTCAGTGCCATTCTCCGT
CAGCCGGCCCATCTCAGTCGCCAAGAGAAACTAGACTACGTTGGTGAAGTCATCAAG
CTACTCGGTATGGAGACGTACGCAGACGCAGTCGTTGGTGTTCCCGGCGAGGGGCTC
AATGTTGAACAGCGCAAGAGGTTAACTATTGGAGTGGAATTGGCTGCAAAACCACAA
CTGTTGTTGTTCCTTGATGAGCCTACCTCTGGTTTGGATTCTCAAACATCGTGGTCCAT
TCTCGATCTCATAGATACCTTAACTAAACACGGCCAGGCCATTCTCTGCACTATTCATC
AGCCTTCGGCTATGTTGTTCCAGCGCTTCGACCGTCTTTTATTCTTAGCTAAGGGTGGA
CGAACAGTGTACTTTGGTGATATTGGTGAAAACTCCTCTATTCTTTCCAGTTATTTTGA
AAGGAACGGCGCCAAGCCTCTATCTCCTGGCGAAAATCCGGCGGAATGGATGTTGGA
TGTGATCGGTGCCGCGCCAGGGTCTCACAGTGATATCGACTGGCCAGCCGTATGGCGT
GAAGGGCCCGAGCGTGTTGCTGTTCGGGAGCATCTTGCTGAACTGAAGGCAACGCTC
TCTGGAAAATCATCCAATGATGATGCAGCCCAGGATGATCCCGGCAGCCTAAAGGAAT
TTGCAGCTCCATTTCATGTCCAGCTATACGAATGTCTCCGCCGCGTCTTTGCACAATAC
TTCCGTACTCCAACTTATTTGTGGTCCAAGTTCGCCCTCTGCGCTCTTTCCGCCATCTA
CATCGGATTCTCTTTCTTCCAAGCCAAGAACAGTCTTCAGGGTCTCCAAAACCAAATG
TTCTCCATCTTCATGCTGATGACCATCTTCGGCAACCTCGTCCAGCAGATCATGCCGC
ACTTCGTAACTCAAAGATCCCTCTACGAAGTCCGCGAGCGTCCCTCCAAAACCTACTC
CTGGCAAGCGTTCATGACCGCCAACATCCTCGTCGAGCTCCCCTGGAACACCCTCAT
GGCCGCCGTCATCTTCTTCTGCTGGTATTATCCCATCGGACTGTACCGTAACGCTGAAC
CCACCCACGCCGTCACCGAGCGCGGCGGCCTCATGTTCCTGCTCATCTGGACCTTCCT
TCTGTTCACCTCCACCTTTGCGCATATGATTATCGCGGGGATTGAATTTGCTGAAACCG
GCGGAAACATCGCCAATTTACTATTCTCTCTCTGTCTGATCTTCTGCGGTGTGCTCGCC
ACCCCTGAATCTCTCCCCGGCTTCTGGATCTTCATGTACCGCGTCTCGCCCTTCACTTA
CCTCGTGTCTGCTATGCTCTCTACCGGTCTCAGCGGGACGACTGTGACTTGCGAAGCG
GTCGAGTATCTGGGCTTTAATCCCCCGGCGAACGAAACCTGTCAGTCCTATATGGCAG
ACTATGTGGCGCTCGCGGGAGGGTATCTGAAGGATCCGTCGGCGACGAGCGGATGTG
AGTTCTGCAGTGTGTCGGGCACGGATACGTTTTTGGCATCGGTGTCGAGCCATTTTAG

TGATGCGTGGAGGAATTTTGGGTTGATGTGGGTTTATATTATCTTTAATATCTTTGCTGC
GGTTTTCATTTATTGGCTTGCTAGGGTGCCAAAGGGGAAACGATCGAAGGTTTGA
>CYP561_HRI11
ATGTCTTCCCGTATCCCCAACCAACAGTATTACCCCAAGCCTCCAGCTGTACGAGTAG
ACGTGTTCAATTCAGCCGCCTTGTTATTTATTCTCGTAAGTCCAAGATGTCGGAACGAT
ACATATCAACACTGAACGATGCACTTCATAGGTTTTGCTTTACCGAGTAGGACTGGTC
TTATATCGGATATATCTTCATCCATTACGTGCAATTCCTGGTCCAAAACTCTGGGCCGTA
ACCGACTTGCCCTACACATATACCGTCGATATTGCAGGGACAGGTGTTCACATTATCGC
CAAGCTCCACAGGAAATACGGTCCTGTTATCCGAATAGGGCCTGACCGTCTTGCTCTA
GATGGTAGCATAGGCTGGCCCCAAGTCTTTGCTCGTCGCACTGATGGAAATGCCGAGT
TTGAAAAAGCACCGCTGTTCTTCCCTCGCGGAACCGAGCAGTCTATAGCATCGGCAC
CTAGAGATGTACACCGTCGGCAACGAAGACAACTTGGGCACGCTTTCAGTGACGCAG
CCATGTATGAGCAGGAAGGTATCCTGAAGAAATACATCCAGCTACTCGTGGATCGCGT
CCAGCAGCATGCAGACTCTGGGACATCAATGAACATCGTTCAATGGCTCAACTTTACC
ACTTTCGACATTATCGGCGATATGACATACGGAGAGTCGTTTGGCTGTCTTGAAAGCA
GCAAATACCATCCATGGGTTCTTAGCGTTTTTGAAGGCATAAAGAGCGATGCCTTTTC
ACGAGCTTGCAGGAAGTATCCTACCATCGGCCCTCTAGTTGAGAAATTATTTGGATCA
AAAAAGGGTGAAGAAAACAATCGTTTGGCCATGGAGAAAGGAAAAGTACGTATGGA
ACTTAGCACGGAGCCAAACGGTCGCCGTGATTTCGTGACTTACATGCAACGACCAAG
TCGCGATGGAACCCCAGGTTTCTCAGACGCCGAGATTATGTCTAATTTACCTGTGCTC
GTTGTTGCCGGCAGTGAAACGACAGCAACCGCATTATCAGGGTTTTTCTTTTACTTGG
GTCAGACACCTCGTGTGAAGGCAAGAGTCATAAATGAGATCCGGAAAGCATTTCAAG
ACGAGTCAGAGATTGATTTGATCAGCACTAATAACCTCGAATACTTGCATGCATCAATT
GAGGAAACTCTGAGAGTATATCCGCCGGCAGGACTTACTCCACCCCGAGCATCACCT
GGTGCTGAGATCGATGGGAAGTTTGTCCCCCGAGGAGTAAGTCGCTCTTTTAGATTGA
CCCCCCCCCCCGGCCCCTCAGAAGCTAAATTGTCAGTAGACCGCGGTTCATGTGATGC
AATGGGCAACATTTCGGAACCCAGAACATTTTCTCGAGCCTGACTCTTTCTGTCCTGA
AAGATGGTTAAAGAATACGCATCCACTATATGATGCCAAGTTCGCCAATGACAATCAC
GAAGTATTCAAACCATTCAGTCATGGCCCTCGCGATTGCATAGGAAAGAACTTGTCCT
ACGCGGAGATGCGACTGATAGTATCTAAACTCTTGTATAGATTTGATTTCGAACTCCTA
GCTGGGCAAGATGATTGGCATGCGACTCAAGCCATATACATAACTTGGGAGAAAGGT
CCACTGTACATAAAGTTCTCTTCCAGAAAACAATGA
>CYP65_HRI11
ATGTTGTCAGTGGTTGCTCTAATAGCAGCCCTCTATCTTCTTTACTCTTTGATTTACATT
ATCCGGGTTCTTTACTACCATCCTCTCAGTCACATCCCTGGTCCGACACTATGGATCGC
TTTTCCAATATTTCGTTACCTCGCGTCGATTCGAGGGAGGCTAGATATCGATCTTCGCG
CTCTCCACCAGAAATATGGTGAGGCTGTTCGATTTTCGCCCGATGAGGTATCCTTCATC
ACCGCCCAAGCCTGGCGGGACATTTATGGCCACGGCCACAAGCAACTGCCTAAGTTC
CAACTCTCAGGTATAAACGGTTTAAATATCATTAGCTCAAATGATATCGACCATACTCG
ATACCGCAAAGCTATATCTCATGCCTTTTCCGCGAAAGGACTTCAAGCGCAAGAGCCG
ATCCTGAAAACATACGTCGATAAACTTATCACCAAATTGGAGCACCCAGCAAAGTCTC
AGTTGCCTATAGATATGGTAATGTGGTATAATCTTACAACATTTGATCTCATCGGCGATC
TTGCCTTTGGTCAATCCTTCGGCGGACTCGAGAACTCACAGTATCACTTCTGGGTCTT
GACAATTTTCCAATCCATACACATACTAGAATTTATCAGACTCAAGGGTATCTATCCTAT
ATTCTCCCAATTGGTCAAACTTTTTATACCCCGTAGTTTGGCAAACGCACGTCAACGG
CAGCTGAACTACAGCAAAACCGCGGTACAAAAGAGGCTTACGAACCATAAGCCCTCC

GACGTTGCAGACTTCATGGATTCCATGCTCCGACATCGCGGCGAGAAAGATAGCCTA
AGTGATGAGGAGCTAGCAGAAAATGCCAGTATTCTTGTCATCGCTGGCAGCGAAACT
ACCGCCACGTTGTTGTCGGGGGTAACCTTCTTACTTATGAAGAACCCAGCGGCGATG
AAAAGGGTAACGGATGAAGTGCGGTCGGTCATGCAAACTGAAGAGGATATCACTTTT
GCAAACACAACTGCAAATCTCCCATACATGTTAGCCTGTCTTGATGAGGCCCTTCGAA
GGTATCCACCGATCCCTACTGGATTGCAACGAGTTACCACTTCGACTACAATTATTTCG
GGCTATCAGGTCCCTCCGAACGTAAGATCAGAAGTGTATCGTTATTCCAGAAAAAGTA
GCTAACAATCCCCAAAGACTAAAGTAGCTGTTCATCAATCCGCGGCGTATCATTCTTC
TCTCAATTTCCACAAGGCCGAGTGCTATATCCCAGAACGCTGGCTCCCAGCAGCGAA
AGACGATCCACAATCGCCATTCTTTAACGATAATCGCGATGTTTTGCAGCCATTCTCTA
CCGGACCACGTAACTGCATCGGCCGAAATCTAGCATATAACGAGATGCGCGTCATCCT
GGCTCGGATAATCTGGAAATTTGACCTTGAATTGTGTCCTGAATCTCAGGCGTGGGAC
GATCAGAAATCATACACTCTGTGGGACAAGCCCCCGCTGATGTGCAAGTTGACACCG
AGGCTATATTGA
>CYP68_HRI11
ATGGAGCCCTTAAAATCTTCAATTGTCCTGTTTCCTAACAGCCTGCCTCCACTTGCCCT
AAACCCTATCACCACTTCGATATTACTTGCCGCTGTACTTGCAATACTTTTTTAAGTA
GGAAATCCAGCAACCTTCCTCATGCCAATCCTCCATCCTGGTTTTCCCCGACAATTATA
ATGCAGTTAGACGCTGGGAAACGCGGCATGGATATCCTGGATGATGGCCGAAAGAGG
TTTCCTGACAGAGCATTCCGAATGATCAACAACAACGGAGAAATAATTGTTCTCCCAT
CGCGTTTTGCGCATGTTATCCGCAACGAAGAAGGCTTGAGCTTTGCCGACTCGACCA
CACGTGTAGGCTCTGTCAATTGTTGTATTCCGAGCCAGAATATACTAATTGTCTAGGAC
TTCCACGCCCAAGTTTCAGGATTCGCACCTGTAGGTGTTATAGGTCACCGAGGACAG
GTATTACAGAATCTTGCTAGAAAGCCACTCACAAAACTTCTAAGTAAGTTTGGTCTTA
GGAACGATTCTACTGAGATACACGGCTAATATGCGTCTGTCTAGATACCGTTACCGAG
CCCCTAGCTTCTGAAGCTACCTTCGCAGTTGATCGTGTCTTAGGTGATTCCACAGGTA
AAGCCTTGAGTCCAGGTAGTCTTTTTGCGATCAACATGATCTAATCTGAAGTAGATTG
GCAAGACACCCTCATTTTGGATTCCATGCTGAACATAATCTCACGTCTTTCGTCGCGT
GTTTTCCTGGGTGACAAACTTTGTCGCAACGAAGATTGGCTCAAGGTCACGAAGGAA
TATACTGTGGAGATGTTGCCAGCGGCATTAAAACTGACGCTCGTGCCCGTTGGCTTTC
GATTTCTCCTTCCCATGTTCCTCAAACAGTACAGAAGCGTTCGTGAACATCTGCGGCG
CTCTCAGGAGCTGATCAATCCCGTAATCGAGGAGCGTCGTGTACTGAAAGAACAAGC
ACGCAGGGCAGGAAAGCCGGTTCCAAAGTTTAATGATGCCATCGAATGGGCCGAAGC
GGAATGCAATGGTGTCAGCTATGATCCTGCCTGTCTGCAACTCATTCTATCATTTGCTG
CCATACACACCACGTCGGACCTTCTGACAAAGATTATGTTGCAATTGGCCAAACAACC
CGATCTCATAGACGCCCTACGGCAAGAAATCATTAGTGTTCTTAAAAAGGATGGATGG
AACAAAACCTCGTTATACAATATGAAGCTGTTGGACAGCGCAATGAAGGAAGCACAG
AGACTGATGCCTAACGAGCAGGGTAAGTCCTTGCTTATCCTGCTTTCCGATACCATTC
AACTGACAATTTGAAGTAAGTATGCACCGGATCGCAACCAAAGATGTACAATTGGCG
GAAGAGAACATCACCATCCACAAGGGGCAGTATGTCGTGGTAGATACCCAGGGTGAG
GACTCAGCCGTGTACGAGAACCCAGAAAAATTCGATATATACCGCTGGTTGCGCTTGC
GAGAAACCCCCGAGTTTGCTAATAAGGCACACTTTGTCAGCACGAGCCCTGCGCACC
TAGGGTTTGGACACGGAATACATGCCTGCCCCGGTCGCTTCTTCGCGGCCAATGAGAT
CAAGATCACACTTTGTTTCCTTCTTCTTAAGTATGACTGGAAATTACCTCCTGGAACA
AATATTAAAATGACTACCTTTGGAACCAGCTACATGCTAGATCCGAGGAGTACTTTGA
GGTTCAAGAGAAGGGAAGCAGAAATTGATTTGGAAGTGTTGAAGTTTGAGTAG
