# Supplementary material for: A Xenobiotic Detoxification Pathway through Transcriptional Regulation in Filamentous Fungi
Source: mBio. 2018 Jul 17;9(4):e00457-18. doi: 10.1128/mBio.00457-18 (PMC6050962; doi:10.1128/mBio.00457-18)
Supplement: TABLE S1 [file mbo004183984st1.pdf]

Table S1. Plasmids and primers used in this study.

Plasmids	Descriptions	Sources
pYHN3-pShEF1α	Overexpression plasmid	Sang et al. (44)
pYHN3-ptrpC	Overexpression plasmid	This study
pYHN3-ptrpC-CYP561	CYP561 overexpression plasmid	This study
pYHN3-ptrpC-CYP65	CYP65 overexpression plasmid	This study
pYHN3-ptrpC-CYP68	CYP68 overexpression plasmid	This study
pYHN3-ptrpC-ShPDR1	ShPDR1 overexpression plasmid	This study
pYHN3-ptrpC-ShatrD	ShatrD overexpression plasmid	This study
Topo-ΔShXDR1	ShXDR1 knock-out plasmid	This study
pYHN3-ptrpC-dsRNAXDR1	ShXDR1 knock-down plasmid	This study
p415-PtrpC-Cas9-TtrpC-CYC1t	Plasmid containing a Cas9 endonulease (Addgene Plasmid #68059)	Matsu-ura et al (50)
p426-SNR52p-gRNA.csr-1.Y-SUP4t	Plasmid containing a gRNA-csr-1 construct (Addgene Plasmid #68060)	Matsu-ura et al (50)
p426-SNR52p-gRNA.ShXDR1.Y-SUP4t	Plasmid containing a gRNA-ShXDR1 construct	This study
Topo-ShXDR1^{M853}	Plasmid with the partial region of ShXDR1^{M853}	This study
pYHN3-MCS	Plasmid with multiple cloning sites	Sang et al. (44)
pYHN3-ShXDR1^{T853}	Plasmid with the upstream (1583 bp) and full length of ShXDR1^{T853}	This study
NeoR-ShXDR1^{T853}	Plasmid with the neomycin resistance gene and upstream (1583 bp) and full length of ShXDR1^{T853}	This study
pYHN3-303bp-pShPDR1	Plasmid with the upstream (303 bp) of ShPDR1	This study
pYHN3-354bp-pShPDR1	Plasmid with the upstream (354 bp) of ShPDR1	This study
pYHN3-479bp-pShPDR1	Plasmid with the upstream (479 bp) of ShPDR1	This study
pYHN3-1289bp-pShPDR1	Plasmid with the upstream (1289bp) of ShPDR1	This study
pPAD80	Plasmid containing HA	Delley and Hall (45)
pYHN3-ptrpC-ShXDR1HA	Plasmid with HA tagged ShXDR1	This study
pYES2-atrD	Galactose induced ShatrD plasmid	This study
pYHN3-ptrpC-TCONS_00005783	Glutathione S transferase overexpression plasmid	This study

Primer Name	Primer Sequence (5'-3')	Descriptions
**Primers for qPCR analysis**		
QF_CYP561<br>QR_CYP561	AGATCGATGGGAAGTTTGTCC<br>GTTCTGGGTTCCGAAATGTTG	Quantify expression of *CYP561* gene
QF_CYP65<br>QR_CYP65	ATTTCCAGATTATCCGAGCCAG<br>GAGTGCTATATCCCAGAACGC	Quantify expression of *CYP65* gene
QF_CYP68<br>QR_CYP68	CAACCCGATCTCATAGACGC<br>GATCCGGTGCATACTTACCTG	Quantify expression of *CYP68* gene

QF_ShXDR1	GCTAGATCACGAGCTTGAAGAA	Quantify expression of *ShXDR1* gene
QR_ShXDR1	GACGACAACATGGAGGATCAA	
QF_10618	TGCATCCAGCGTTCTAGTATTC	Quantify expression of TCONS_00010618
QR_10618	CGAACCAGCAGGTTGTTAGTA	
QF_3057	AATGGCGGGAGTCTGTTG	Quantify expression of TCONS_00003057
QR_3057	TCTGATCCAATGCCCAATTCT	
QF_2727	TCTGCAGTCCGCAACTTATT	Quantify expression of TCONS_00002727
QR_2727	GGGATCCGATTCATCTGTCTTC	
QF_23546	CTTCTACCACCACCCTTTCTAC	Quantify expression of TCONS_00023546
QR_23546	TCGACATCAGCACCGATTATAC	
QF_5783	GCTCGAGGAGCTTAAAGAAGT	Quantify expression of TCONS_00005783
QR_5783	AGATGGGAATTCGACCATTAGG	
QF_YFP	GCACAAGCTGGAGTACAACTA	Quantify expression of YFP
QR_YFP	TGTTGTGGCGGATCTTGAA	
QF_PDR1ChIP	GTGTTCGATTGTGTTCGATTGT	Quantify *ShPDR1* promoter enrichment
QR_PDR1ChIP	CTCTCCCATTCCACGGTATTATT	
QF_CYP561ChIP	GATGCTGCGTTCTGGTTATTG	Quantify *CYP561* promoter enrichment
QR_CYP561ChIP	GCGGCAGCACAAACTTAAA	
QF_Bcactin	CTGAGCGTGGTTACACTTTCT	Quantify expression of *Bcactin* gene
QR_Bcactin	TGACTGGCGGTTTGGATTT	
QF_BcatrD	TTCCGGTCTATTGCTTCACTATC	Quantify expression of *BcatrD* gene
QR_BcatrD	GGGACCGCAAATCCAGTATAA	
QF_BcCYP65	GCTATGTCTCATGCCTTCTCTG	Quantify expression of *BcCYP65* gene
QR_BcCYP65	TCGCTTCGTGCTCCAATTA	
**Primers for generation of different types of mutants**		
F_ApaI_ ptrpC	ATTTGGGCCCCGTTAACTGATATTGAAGGA	Amplification of ptrpC from pYHN3 vector
R_NcoI_ ptrpC	ATTCCCATGGTTGGATGCTTGGGTAGAATA	
F_KpnI_CYP561	ATGTGGTACCATGTCTTCCCGTATCCCCAA	Amplification of *CYP561* gene
R_SpeI_CYP561	GCGCACTAGTTCATTGTTTTCTGGAAGAGAACTTTATGTAC AGTG	
F_NcoI_CYP65	ATGTCCATGGATGTTGTCAGTGGTTGCTCT	Amplification of CYP65 gene
R_SacI_CYP65	ATGTGAGCTCTCAATATAGCCTCGGTGTCA	
F_KpnI_CYP68	ATGCGGTACCATGGAGCCCTTAAAATCTTCAATTGTCCTG	Amplification of CYP68 gene
R_NotI_CYP68	ATGCGCGGCCGCCTACTCAAACTTCAACACTT	
F_KpnI_ShPDR1	ATGCGGTACCATGTCTTTATTAGGGAATCTCAACCCTAAT	Amplification of *ShPDR1* gene
R_SpeI_ShPDR1	ATGCACTAGTTCAAACCTTCGATCGTTTCCC	
F_SacI_ShatrD	ATGCGAGCTCATGGCGACCTCGACAC	Amplification of *ShatrD* gene
R_NotI_ShatrD	ATTTGCGGCCGCCTATTTCTTCCTAAACCACT	
R_XhoI_ShatrD	ATGCCTCGAGCTATTTCTTCCTAAACC	
F_SacI_upXDR1	ATAAGAGCTCTGCCATGCGAATAAGGA	Amplification of upstream of *ShXDR1*
R_SpeI_upXDR1	ACGCACTAGTCACTAATTATCGCATAGTAT	

F_NotI_downXDR1<br>R_ApaI_downXDR1	ATTTGCGGCCGCGAGTTCGGTTTTAGCA<br>ATACGGGCCCACTCTTCGCATTCTCGTCC	Amplification of downstream of *ShXDR1*
F_upXDR1<br>R_YG	TGCCATGCGAATAAGGAGATGTTCG<br>GGATGCCTCCGCTCGAAGTA	Amplification of the upstream of *ShXDR1* and split marker
F_HY<br>R_downXDR1	CGTTGCAAGACCTGCCTGAA<br>ACTCTTCGCATTCTCGTCCCGGTTC	Amplification of the downstream of *ShXDR1* and split marker
F_ApaI_upXDR1<br>R_NotI_ShXDR1	ATTTGGGCCCTTGTCATCTCTTACGGGAAT<br>ATGCGCGGCCGCTTACATTCCACCTTGATGTTG	Amplification of the 1581bp of upstream region and full length of *ShXDR1*
F_SacI_XDR1SNP<br>R_SpeI_XDR1SNP	ATAAGAGCTCGATTGGTTATATGTTAGACAAGG<br>ACGCACTAGTTTACATTCCACCTTGATGTTG	Amplification of the partial sequences of *ShXDR1*
F_XDR1SNP	GATTGGTTATATGTTAGACAAGGATATCTG	Amplification of the partial sequences of *ShXDR1* and split marker with R_YG
F_det_upXDR1	CACTGAAGTCGATGGCGTCTATCTA	Amplification with R_YG to confirm HRI11(ΔShXDR1)
R_det_downXDR1	TCCGTCAACCTAACCATGCC	Amplification with F_HY to confirm HRI11(ΔShXDR1) and HRI11 ($ShXDR1^{M853}$)
F_ KpnI_00005783<br>R_NotI_00005783	GCCGGGTACCATGTCTTCCAGTTTCTACCTTG<br>ATTTGCGGCCGCCTACTTGGCCTGCCATTTCT	Amplification of TCONS_00005783
F_AscI_dsXDR1<br>R_SwaI_dsXDR1	ATACGGCGCGCCCCCCACCACAGATCTTCGGGG<br>GGCCATTTAAATCGTACAAGCGGGTAGTTTTCCATC	Amplification of the sense of ShXDR1 fragment
F_BamHI_dsXDR1<br>R_SpeI_dsXDR1	ATCGGGATCCCGTACAAGCGGGTAGTTT<br>ATGCACTAGTCCCCACCACAGATCTTCG	Amplification of the antisense ShXDR1 fragment
F_KpnI_ShXDR1<br>R_BamHI_ShXDR1	ATGCGGTACCATGATGGACTCCATGCATCGTGAAAGGAAT<br>ATGCGGATCCCATTCCACCTTGATGTTGCA	Amplification of ShXDR1 without stop codon
R_NotI_ShXDR1HA	ATTTGCGGCCGCTTAAGCGTAATCTGGAACGTCATAT	Amplification of HA tagged ShXDR1 with F_KpnI_ ShXDR1
F_SacI_1289upPDR1<br>F_SacI_479upPDR1<br>F_SacI_354upPDR1<br>F_SacI_303upPDR1<br>R_NcoI_upPDR1	ATTTGAGCTCGAGGAGGAGCTTCGTGGAGC<br>AGCGGAGCTCTGCATATGAAGTTGTTCTAT<br>ATCGGAGCTCTGGAATGGGAGAGTTCCTC<br>ATCGGAGCTCTGGAAATGGGCACCAGAAAT<br>AGCGCCATGGGATGTGCTAGTCAGAGGATA	Amplification of different lengths of ShPDR1 promoter

F_NheI_SNR52p	TTGGGCTAGCGGTAAAGGTGCGCATTTTTT	To insert gRNA-ShXDR1 target sequence into the vector containing gRNA constructs
R_gRNA_XDR1	ACATACCCAGGTCTGTTCGCGATCATTTATCTTTCACTGC	
F_gRNA_XDR1	GCGAACAGACCTGGGTATGTGTTTTAGAGCTAGAAATAGC	
R_KpnI_CYC1t	ATTGGGTACCGGCCGCAAATTAAAGCC	
**Primers for amplification and sequencing of partial sequence of *ShXDR1* gene**		
F1_ShXDR1	ATTGTGACCTCACGAAGAGTTGTA	
R1_ShXDR1	TCATATCAGAAAGTGCTTCTACCG	
F2_ShXDR1	CACATCTCCGGAACAAAGTACC	
R2_ShXDR1	CGATACTATGCGATAACCGAATG	
F3_ShXDR1	GCCACCTAGTACCAAAAGATGAAG	
R3_ShXDR1	TCACTAATGCAGAAACAGGAAAGT	
F4_ShXDR1	TCGACTTTCCAATTATGCTATTCA	
R4_ShXDR1	GAGTTTCTAGTAAATGCGCCTCA	
